# St’át’imcets frustratives as not-at-issue modals

**DOI:** 10.1515/ling-2020-0211

**Published:** 2022-08-12

**Authors:** Henry Davis, Lisa Matthewson

**Affiliations:** Department of Linguistics, University of British Columbia, Totem Field Studios, 2613 West Mall, Vancouver, BC V6T1Z4, Canada

**Keywords:** epistemic modality, frustratives, non-culminating accomplishments, prospective aspect, Salish

## Abstract

This paper provides an analysis of the ‘frustrative’ marker *séna7* in St’át’imcets (Lillooet Salish), and compares it to similar elements cross-linguistically. *Séna7* appears in a range of discourse contexts, including when events have an unexpected outcome, fail to continue, or fail to take place optimally. We argue that *séna7* felicitously applies to a proposition *p* only if there is a salient true proposition *q* and the speaker did not expect *p* and *q* to both be true. *Séna7* encodes epistemic modality, refers only to the speaker’s epistemic state (ignoring the common ground), and has no effect on at-issue truth conditions (*séna7(p)* entails *p*)*.* We show that *séna7* provides a diagnostic for distinguishing between entailments and implicatures in the language, and a clear diagnostic for the distinction between futures and prospective aspects. We compare *séna7* with similar elements in Tohono O’odham, Kimaragang and Tagalog. We argue that *séna7* and the Kimaragang frustrative can be captured by the same analysis once independent features of their tense/aspect systems are taken into account. Following Kroeger (2017. Frustration, culmination and inertia in Kimaragang grammar. *Glossa: A Journal of General Linguistics* 2(1). 56. 1–29), but pace Copley and Harley (2014. Eliminating causative entailments with the force-theoretic framework: The case of the Tohono O’odham frustrative cem. In Bridget Copley & Fabienne Martin (eds.), *Causation in grammatical structures* (Oxford Studies in Theoretical Linguistics 52), 120–151. Oxford: Oxford University Press), we argue that frustratives should not be unified with non-culminating accomplishments, and can be analyzed without appealing to causality or efficacy.

## 1 Introduction

### 1.1 Overview

This paper is a contribution to the growing literature on so-called ‘frustrative’ elements, focusing on data from St’át’imcets (Lillooet Salish). Frustratives have been defined by Overall (2017: 479) as in (1), based on his study of 54 Amazonian languages.

(1)

**Table:** 

Frustrative is a grammatical marker that expresses the nonrealization of some expected outcome implied by the proposition expressed in the marked clause.

Overall notes (2017: 479) that there has been ‘little typological analysis of this category’. Until recently, this was also true of formal analyses; recent formal approaches include Copley (2005), Copley and Harley (2014), Kroeger (2017) and Carol and Salanova (2017).

Introductory examples of the St’át’imcets frustrative *séna7* are provided in (2)–(4). *Séna7* appears, for example, when events have an unexpected outcome (2), fail to continue (3), or fail to take place in an optimal fashion (4).1St’át’imcets data are given in the van Eijk orthography employed throughout St’át’imc territory: see van Eijk (1997) for a conversion chart to the North American Phonemic Alphabet. The symbol *7* represents a glottal stop.Morpheme glosses follow the Leipzig Glossing Rules, with the following additions: a = paragogic “a”, abs = absent, act = active intransitive, adhort = adhortative, aia = ability/involuntary action, anti = antithetical, aut = autonomous intransitive, circ = circumstantial modal, cntr = contra expectation, cre = consonant reduplication, deic = deictic, des = desiderative, dir = directive transitivizer, emph = emphatic, epis = epistemic modal, exis = existential, fre = final reduplication, inc = inchoative, ind = indirective applicative, indep = independent pronoun, mid = middle intransitivizer, ntl = neutral, nts = non-topical subject, ooc = out-of-control, prep = preposition, prosp = prospective aspect, prsup = presupposed, rep = reportative, rlt = relational transitivizer, stat = stative, vis = visible. Clitic boundaries are indicated by an equals sign (=) and reduplicants are separated by bullets (•).

(2)

**Table:** 

*Ka-mág-a=ku7*	***séna7**,*	*t’u7*	*áy=t’u7*	*kw=s=7áts’x-n-as.*
circ-bright-circ=rep	**sÉna7**	but	neg=excl	det=nmlz=see-dir-3erg
‘It got brighter, but he still couldn’t see it.’			
(Charlie Mack, in Davis 2017)

(3)

**Table:** 

*Say’sez’=lhkán=tu7*	***séna7**,*	*t’u7*	*cw7aoz*	*aylh*	*kwenswá*	*sáy’sez’.*
play=1sg.sbj=dist	**sÉna7**	but	neg	now	det+1sg.poss+nmlz+ipfv	play
‘I was playing, but I’m not playing now.’

(4)

**Table:** 

*Wa7*	*aylh*	*ka-7áts’x-m-a*	***séna7**,*	*t’u7*	*cw7áoy=t’u7*	*kw=s=7áma.*
ipfv	then	circ-see-mid-circ	**sÉna7**	but	neg=excl	det=nmlz=good
‘Then he could indeed see, but not very well.’				
(Beverley Frank, in Davis 2017)				

This paper provides a unified analysis of *séna7* which captures all its effects. We propose that *séna7* can be felicitously applied to a proposition *p* only if the discourse context contains a salient true proposition *q* and the speaker did not expect *p* and *q* to both be true at the same time. The analysis entails the following claims about *séna7*: (i) it is inherently context-dependent, since it depends on a proposition provided by the context; (ii) it encodes epistemic modality; (iii) it refers only to the speaker’s epistemic state (it does not place any restriction on the common ground); (iv) it has no effect on the at-issue truth conditions: an utterance of *séna7(p)* asserts *p.*

We show that *séna7* provides a clean diagnostic for distinguishing between entailments and implicatures in the language. This enables us to confirm the difference between predicates which only implicate, as opposed to entail, culmination in the perfective aspect. It furthermore provides a clear diagnostic for a temporal distinction which can otherwise be difficult to tease apart in general, in any language: the distinction between futures (which place the evaluation time before the reference time), and prospective aspects (which place the reference time before the event time).

In the last part of the paper we address the relation between *séna7* and other similar elements crosslinguistically including the Tohono O’odham frustrative *cem* (Copley 2005; Copley and Harley 2014; Hale 1969), the Kimaragang frustrative *dara* (Kroeger 2017), and the Tagalog ability/involuntary action marker (Alonso-Ovalle and Hsieh 2017a, 2017b, 2018). We argue that in spite of apparent empirical differences between St’át’imcets *séna7* and the Kimaragang frustrative, the two elements can be captured by an identical analysis, with the differences deriving from independent features of the tense/aspect systems of the languages*.* In the debate between Copley and Harley (2014) and Kroeger (2017) over the best analysis of frustratives, we find evidence in support of Kroeger’s view: frustratives should not be (even partially) unified with non-culminating accomplishments, and frustratives can be analyzed using standard modal tools without needing the additional notions of causality, forces, or efficacy.

In the remainder of this section we provide background information on the language, *séna7,* and our methodology. In Section 2 we show how our proposal works by providing a systematic overview of the interpretations of *séna7-*clauses with different Aktionsarten. Section 3 presents our formal analysis and explores more detailed predictions: for example, we show that *séna7* is speaker-oriented, not at issue, and does not induce a causality effect. Section 4 shows how *séna7* interacts with, and distinguishes between, the two grammaticized forms of future time-reference in St’át’imcets, and argues that *séna7* acts as a semantic diagnostic for prospective aspect within the class of motion verbs. In Section 5 we compare *séna7* to other frustrative elements and their analyses crosslinguistically. Section 6 concludes and points to future research directions.

### 1.2 Background on St’át’imcets and on *séna7*

St’át’imcets, also known as Lillooet (ISO 639-3 lil), is a Northern Interior Salish language spoken in the southwest interior of British Columbia, Canada. It is highly endangered, with fewer than 100 first-language speakers at the time of writing (Dunlop et al. 2018). All unattributed examples in the paper come from original fieldwork by the authors. In cases where the data is taken from published narratives, the speaker/storyteller is identified by name.

*Séna7* is one of a small closed class of lexical adverbs in St’át’imcets; these adverbs generally occur after the first predicative element of a clause (including any enclitics).2Crosslinguistically, elements with frustrative semantics instantiate a range of different grammatical categories. We return to this briefly in Section 6. Unlike enclitics, *séna7* is prosodically independent and may also occur clause-finally or – less frequently – in other post-predicative positions. We do not address its clause-internal distribution in detail here, but we assume that it always takes sentential scope.

*Séna7* can appear in both monoclausal and biclausal structures. Biclausal cases were given in (2)–(4) above, and a monoclausal case is shown in (5).

(5)

**Table:** 

Context: Someone is trying to sell you something but you don’t want it (you have money but you don’t want to spend it).								
*Wá7=lhkan*	***séna7***	*es=qláw’.*
ipfv=1g.sbj	**cntr**	have=money
‘I have money (but I won’t spend it).’								

We will argue that semantically, *séna7* always relates two propositions, but one of them can be either contextually provided or accommodated.

*Séna7* has previously been glossed as ‘though’ (van Eijk 1997), ‘counter-to-expectation’ (Davis 2017), ‘often untranslatable; expresses an unfulfilled condition, a change of mind or some other contradiction or contrast’ (van Eijk 2013), and as ‘against expectations (either the speaker’s, the hearer’s, or somebody else’s); often difficult to translate into English’ (Alexander et al. in prep.). These informal characterizations give something of the flavor of *séna7*, but do not offer full insight into its semantic or pragmatic contribution. The first attempt at formal analysis of *séna7* was by Davis and Matthewson (2016); the current paper builds on and extends the proposals made there.

We will henceforth gloss *séna7* as cntr, for ‘contra expectation’.

### 1.3 Methodology

Several data collection methodologies were employed in this study. Primarily, we conducted targeted elicitation using standard semantic fieldwork methods involving controlled discourse contexts (see, for example, papers in Bochnak and Matthewson 2015; Krifka 2011; Matthewson 2004b; Tonhauser and Matthewson 2016). In addition to the usual methods of eliciting acceptability judgments and translations in context, we utilized two less common techniques as a response to the radical context-dependence of *séna7*. First, we sometimes provided the consultants with a sentence containing *séna7* and asked them to provide a suitable discourse context in which the sentence could be uttered. Second, we conducted a variant of the ‘cloze’ test familiar from language acquisition studies: we provided the speakers with a clause containing *séna7*, and asked them to provide a felicitous completion (i.e., a follow-up clause). Instances of this elicitation method are marked with ‘…’ between the first and second clauses. Thus, wherever the data includes a ‘…’, the material after the dots was volunteered by the consultant.

Finally, we checked our generalizations against all instances of *séna7* in five text collections (Alexander 2016; Edwards et al. 2017; van Eijk and Williams 1981; Matthewson 2005; Mitchell in press), as well as all the example sentences in a forthcoming comprehensive English-Upper St’át’imcets dictionary (Alexander et al. in prep). We also examined the large number of instances of *séna7* which have arisen in our elicited data over the years, many of them spontaneously offered in contexts where we were targeting other grammatical phenomena.

## 2 How *séna7* works: case studies of Aktionsarten

Our proposed analysis is given semi-formally in (6).

(6)

**Table:** 

[[ *séna7 (p)* ]]^c^ is felicitous if *c* contains a salient true proposition *q* and the speaker does not expect *p* and *q* to both be true.
If felicitous, [[ *séna7 (p)* ]]^c^ = [[ *p* ]]^c^.

According to this proposal, *séna7* does not affect the truth conditions of its prejacent proposition; instead, it imposes a condition on the relation of the prejacent to another salient proposition (explicit or implicit) within a discourse context.3
Overall (2017: 479) similarly claims (following Adaskina 2005) that frustratives imply two propositions, and that only one of them must be explicitly provided. However, the status of the second proposition is different in the two approaches. For Overall/Adaskina, the implicit proposition *q* is the expected outcome of the prejacent *p,* and the frustrative expresses the non-realization of *q*. In our analysis, the second proposition *q* is a *true* proposition, which is unexpected given *p*. This more flexible approach to *q* allows us to capture the full range of interpretations of *séna7,* as outlined in this and following sections.


In order to show how our appoach to *séna7* works, in this section we offer a systematic exploration of its effects on lexical aspectual classes (Aktionsarten). We will show that the proposal summarized in (6) successfully unifies all of *séna7*’s empirical effects. For background on lexical aspectual classes, see Filip (2012, 2020 and references therein.

### 2.1 *Séna7* with states and activities

Atelic predicates in St’át’imcets – states and activities – show the following interpretations with *séna7:* (i) some expected outcome of the eventuality fails to hold; (ii) the eventuality fails to continue; (iii) the eventuality unexpectedly co-occurs with another one; (iv) the eventuality does not happen ‘well’ or successfully.

Unexpected outcomes of stative eventualities are illustrated in (7)–(11). Notice that the contextually salient proposition *q* may be provided by the second clause of the utterance itself (as in (7)–(9)), or not (as in (10)–(11)). In cases where *q* is not obvious from the utterance itself, we indicate it below the data.

(7)

**Table:** 

*S-qacw*	***séna7***	*ta=n-q’íl’q=a,*	*t’u7*	*wá7=lhkan=t’u7*
stat-break	**cntr**	det=1sg.poss-chair=exis	but	ipfv=1sg.sbj=excl
*ka-mitsa7q-mín-a.*					
circ-sit-rlt-circ					
‘My chair is broken, but I can still sit on it.’

(8)

**Table:** 

*Áma=t’u7*	***séna7***	*ti=wá7*	*zayten-mín-as*	*ti=cúz’a*	meeting,					
good=excl	**cntr**	det=ipfv	business-rlt-3erg	det=prosp=exis	meeting					

*t’u7*	*ícwlh=t’u7*	*[ti=s=]ka-t’ák=s-a.*				
but	different=excl	[det=nmlz=]circ-go=3poss-circ					
‘What she had done for the meeting was good, but it went quite differently.’

(9)

**Table:** 

*Zwát-en=lhkan*	***séna7***	*kw=s=cuz’*	*kwis …*	*mes=kán=t’u7*	*tsicw*
know-dir=1sg.sbj	**cntr**	det=nmlz=prosp	rain	but=1sg.sbj=excl	get.there
*mám’teq.*					
go.for.walk					
‘I knew it was going to rain … but I went for a walk anyway.’

(10)

A:

**Table:** 

*Cúz’=lhkacw=ha*	*saotatíh-am?*
prosp=2sg.sbj=q	Saturday-mid
‘Are you going out partying this weekend?’

B:

**Table:** 

*Ícwa7=lhkan*	***séna7***	*es=qláw’.*			
without=1sg.sbj	**cntr**	have=money				
‘I don’t have any money.’					
Consultant’s comment: “I guess you’re going, even though you’re broke.”

p:

**Table:** 

*I don’t have money* *q:* *I’m going partying*

(11)

**Table:** 

Context: A has to write a paper. The sun is shining, the birds are singing. A says:					
*O,*	*xát’-min’=lhkan*	***séna7***	*kw=n=nas*	*ex•éx•ts*	*áku7*
oh	want-rlt=1sg.sbj	**cntr**	det=1sg.poss=[nmlz=]go	lie•cre•	deic
* (l=ti=)skwél’=a.*				
(prep=det=)sun=exis				
‘I really want to go and lay out in the sun for a while.’					
*p:* *I want to lie in the sun* *q:* *I won’t go and lie in the sun*					

For (11) and other similar cases, we assume that the expected outcome of a mental attitude of desire is that the desired situation obtains. Copley and Harley (2014) achieve a similar effect through their Law of Rational Action, which states that a volitional agent with a desire will act as a force which ceteris paribus will result in the desired situation coming about.

Like states, activity predicates also appear with *séna7* when some expected outcome of the event fails to happen. Examples are given in (12)–(16).

(12)

**Table:** 

*Píxem’=wit*	***séna7***	*áku7*	*sqwém=a,*	*t’u7*	*áy=t’u7*
hunt=3pl	**cntr**	deic	mountain=exis	but	neg=excl
*kw=s=7ats’x-en-ítas*			*ku=ts’í7.*			
det=nmlz=see-dir-3pl.erg			det=deer		
‘They went hunting in the mountains, but they didn’t see any deer.’

(13)

**Table:** 

*Lán=lhkan*	*aylh*	***séna7***	*k’wzús-em …*	*t’u7*	*ay*	*s=xaq’-en-tsálem.*
already=1sg.sbj	now	**cntr**	work-mid	but	neg	nmlz=pay-dir-1sg.pass
‘I’m already working … but I’m not getting paid.’

(14)

**Table:** 

*It’-em=lhkán=t’u7*	***séna7***	*l=ti=s-gáw’-p=a …*	*t’u7*	*áoy=t’u7*
sing-mid=1sg.sbj=excl	**cntr**	prep=det=nmlz-meet-inc=exis	but	neg=excl
*swat*	*ku=k’alán’-min’-ts-as.*		
who	det=listen-rlt-1sg.obj-3erg		
‘I sang at the gathering … but nobody listened.’

(15)

**Table:** 

*T’ák=kan*	***séna7***	*k’ák’em-l’ec,*	*nilh*	*n=s=hul’qs,*
go.along=1sg.sbj	**cntr**	sneak-aut	cop	1sg.poss=nmlz=sneeze
*q’áy-lec=tu7*	*aylh*	*na=ts’í7=a.*		
run.away-aut=dist	now	abs.det=deer=exis		
‘I was sneaking along but then I sneezed, so the deer took off.’				
(Alexander et al. in prep.)

(16)

**Table:** 

*Míts’-lec*	***séna7***	*t’u7*	*ka-túp-ts-s=kan-a.*
duck-aut	**cntr**	but	circ-punch-mouth-caus=1sg.sbj-circ
‘He ducked but I managed to punch him in the mouth.’			
(Alexander et al. in prep.)			

Sometimes, the expected outcome of a state or activity is simply that it continues, so *séna7* flags the fact that the eventuality no longer holds.4
Overall (2017: 481) argues that failure of an event to continue does *not* count as an unrealized expectation. For example, he claims that in (i), ‘the speaker obviously did not expect that the cigarette would not end.’

(i)

**Table:** 

*ui*	*‘hu-**le**-hỹ-ki*
tobacco	smoke- **frust**-nmlz-decl
‘He was smoking (but the cigarette ended) unfortunately.’				
(Kwaza; Overall 2017: 481, citing Van der Voort 2000: 405)

However, it is common for frustratives to mark the failure of an eventuality to continue, either with or without an extra evaluative implication such as is suggested by the translation of (i). A unified analysis of frustratives which relies on the notion of unrealized expectations will therefore only be successful under the assumption we make here, that failure to continue ‘counts’ as unexpected. Cf. also Copley’s (2005) use of inertia worlds. This is shown in (17)–(18) for states, and in (19)–(21) (and (3) above) for activities.5A reviewer pointed out a potential connection to Cable’s (2017) discussion of the Tlingit decessive, which gives rise to cessation inferences with stative predicates. Cable argues that the decessive is simply an optional past tense, and its cessation inferences are conversational implicatures that derive from its optionality. He further proposes that all similar past markers crosslinguistically will be analyzable in a parallel fashion, including the Tohono O’odham frustrative *cem* as discussed by Copley (2005).
*Séna7* shares with the Tlingit decessive the possibility of cessation inferences with statives, as in (17)–(18), as well as the ability for cessation to be absent, as in (7)–(11). However, Cable’s analysis does not apply to *séna7*. Unlike the decessive, *séna7* does not contain past tense semantics, as shown by its appearance in present- and future-tensed clauses (e.g., (10)–(11)). Therefore, cessation cannot be derived from pastness, as in Cable’s account. Moreover, the contribution of *séna7,* which has to do with unexpectedness rather than pastness, is not cancelable and therefore is not a conversational implicature. This is shown for example in (32)–(37) below.

(17)

**Table:** 

*Wá7=lhkan=tu7*	***séna7** ka-táns-a*	*i=wán*	*twiw’t,*
ipfv=1g.sbj=dist	**cntr** circ-dance-circ	when.pst=ipfv+1sg.sbjv	youth

*lán=t’u7*	*ao*	*kwas*	*áma*	*i=n-sq’wáxt=a*	*lhkúnsa.*	
already=excl	neg	det+nmlz+ipfv+3poss	good	pl.det=1sg.poss-leg=exis	now	
‘I used to be able to dance, but my legs don’t work well anymore.’					
*p:* *I used to be able to dance*			*q:* *I can no longer dance*

(18)

**Table:** 

*Qlíl=lhkan=tu7*	***séna7**,*	*t’u7*	*cw7aoz*	*aylh*	*kwenswá*	
angry=1sg.sbj=dist	**cntr**	but	neg	now	det+1sg.poss+nmlz+ipfv	
*qlil.*						
angry						
‘I was angry, but now I am not angry.’

(19)

**Table:** 

*Wá7=lhkan*	***séna7***	*alkst,*	*t’u7*	*kaw-an-tsálem.*
ipfv=1sg.sbj	**cntr**	work	but	far.away-dir-1sg.pass
‘I was working, but I got fired.’

(20)

**Table:** 

*Q’ets’-en-ás*	***séna7***	*kw=s-Jane*	*ta=tsepíts’7=a,*	*t’u7*	*plan*	*tsukw:*
knit-dir-3erg	**cntr**	det=nmlz-Jane	det=sweater=exis	but	already	stop
*ts’ék=tu7*	*na=yáon-s=a.*				
all.gone=dist	abs.det=yarn-3poss=exis				
‘Jane was knitting a sweater, but she stopped: her yarn ran out.’						

*Séna7* also appears on states and activities when the issue is not a failed outcome, but simply an unexpected co-occurrence with another eventuality. In (21), singing a sad song does not cause one to be unhappy, and in (22), having a bath does not cause one to wash one’s hair. It is simply that these two pairs of eventualities usually co-occur, so the co-occurrence of the opposite is unexpected.

(21)

**Table:** 

*N-qwnúxw-alhts’a7*	***séna7***	*[ta]=s-7ít’-em-s=a*	*s-Mary,*	*t’u7*
loc-sick-inside	**cntr**	[det]=nmlz-sing-mid-3poss=exis	nmlz-Mary	but
*áma ta=scwákwekw-s=a.*			
good det=heart-3poss=exis			
‘Mary’s song/singing was sad, but she was happy.’

(22)

**Table:** 

*Sácw-em=lhkan*	***séna7***	*i=n’án’atcw=as,*	*t’u7*	*áy=t’u7*
bathe-mid=1sg.sbj	**cntr**	when.pst=morning=3sbjv	but	neg=excl
*kw=ka-ts’áw’-s-an-a i=n-máqin=a.*				
det=[nmlz=]circ-wash-caus-1sg.erg-circ pl.det=1sg.poss-hair=exis				
‘I had a bath this morning, but I wasn’t able to wash my hair.’				

Finally, states and activities allow *séna7* in contexts where the eventuality does not happen successfully or very well. This is illustrated in (23)–(27). The English translation often includes ‘try’, but this is not literal; it is an attempt by the speakers to render the ‘not very well’ effect.

(23)

**Table:** 

*Zewát-en=lhkan*	***séna7***	*kw=s-Sarah,*	*t’u7*	*cw7áoy=t’u7*
know-dir=1sg.sbj	**cntr**	det=nmlz-Sarah	but	neg=excl
*kwas*		*áma.*			
det+nmlz+ipfv+3poss		good			
‘I know Sarah, but not very well.’

(24)

A:

**Table:** 

*Wa7*	*kán-em*	*k=Marion?*
ipfv	do.what-mid	det=Marion
‘What is Marion doing?’

B:

**Table:** 

*Lhk’wál’us=t’u7*	***séna7**.*
make.baskets=excl	**cntr**
‘I THINK she’s making a basket/She’s trying to make a basket.’	
Consultant’s comments: “She’s not really”; “Probably just learning.”

(25)

**Table:** 

*Ít’-em=t’u7*	***séna7***	*k=Henry.*
sing-mid=excl	**cntr**	det=Henry
‘Henry tried to sing.’

(26)

**Table:** 

*Ít’-em=lhkan,*	*siq’úta=lhkan*	*t’it*	***séna7**.*
sing-mid=1sg.sbj	dance=1sg.sbj	also	**cntr**
‘I sang, and I also danced.’			
Consultant’s comment: “Okay, if you didn’t really know how to *siq’úta* [dance].”

(27)

**Table:** 

*Wá7=t’u7=ti7*	***séna7***	*wa7*	*lam-áy’lh.*
ipfv=excl=dem	**cntr**	ipfv	comfort-child
‘He is trying to comfort the child.’			
(adapted from Alexander et al. in prep.)			

Because the prejacent can contrast in various ways with another true proposition, it is easy to find minimal sets with identical *séna7-*clauses, but different *q*s. This confirms the context-dependence of *séna7.* One such minimal pair is (28)–(29): in (28), the speaker contrasts their earlier hunger with the failure of the hunger to continue, while in (29), the hunger contrasts with the failure to eat.

(28)

**Table:** 

*Tayt=lhkán=tu7*	***séna7,***	*t’u7*	*cw7aoz*	*aylh*	*kwenswá*	*tayt.*
hungry=1sg.sbj=dist	**cntr**	but	neg	now	det+1sg.poss+nmlz+ipfv	hungry
‘I was hungry but I’m not hungry now.’

(29)

**Table:** 

*Tayt=lhkán=tu7*	***séna7**,*	*t’u7*	*cw7aoz*	*kw=n=s=7ílhen,*	*cw7aoz*
hungry=1sg.sbj=dist	**cntr**	but	neg	det=1sg.poss=nmlz=eat	neg
*kwas*		*áma i=s-7ílhen=a*	*láta7*	*q’7-álhcw=a.*	
det+nmlz+ipfv+3poss		good pl.det=nmlz-eat=exis	deic	eat-place=exis	
‘I was hungry, but I didn’t eat – that restaurant doesn’t have good food.’					

Another pair is (30a) and (30b): an earlier state of wanting is contrasted either with the failure of the wanting to continue, or with the failure of the wanted event to be realized.

(30)

**Table:** 

*Xát’-min’=lhkan*	***séna7***	*kw=n=s=7úxwal’*	*i=kel7=án*
want-rlt=1sg.sbj	**cntr**	det=1sg.poss=nmlz=go.home	when.pst=first=1sg.sbjv
*t’iq*			
get.here			
‘I wanted to go home when I first came,

a.

**Table:** 

…	*t’u7*	*cw7aoz*	*aylh*	*kwenswá*	*uxwal’-ál’men.*
	but	neg	then	det+1sg.poss+nmlz+ipfv	go.home-des
‘but I don’t want to go home now.’

b.

**Table:** 

…	*t’u7*	*cw7aoz*	*kw=s=celhcalh-tumcál-itas.*
	but	neg	det=nmlz=allow[-caus]-1sg.obj-3pl.erg
‘but they didn’t allow me to.’

An activity pair is given in (31a), and (31b). The interpretations are respectively ‘event in vain’ and ‘not very well’.

(31)

**Table:** 

*Q’weláw’-em=lhkalh*	***séna7***	*ku=stsáqwem …*
pick-mid=1pl.sbj	**cntr**	det=saskatoon
‘We picked saskatoonberries …’

a.

**Table:** 

*t’u7*	*áy=s=t’u7*	*kwas*	*q’wel.*
but	[nmlz=]neg=3poss=excl	det+nmlz+ipfv+3poss	ripe
‘but they weren’t ripe.’

b.

**Table:** 

*t’u7*	*áy=s=t’u7*	*kwas*	*cw7it.*
but	[nmlz=]neg=3poss=excl	det+nmlz+ipfv+3poss	many
‘but we didn’t get many.’

So far we have only given positive data – environments where *séna7* is felicitous – which do not yet prove that *séna7* itself is contributing the relevant interpretation. Negative data are given in (32)–(37).6Outright rejections of *séna7,* although attested as shown here, are relatively rare because speakers can usually accommodate *some* proposition *q* which contrasts with the prejacent and makes *séna7* acceptable. This is observed also by Alonso-Ovalle and Hsieh (2017b) for Tagalog ability/involuntary action morphology, “The contribution of AIA morphology is elusive because this context-sensitive modal component is easy to accommodate.”A striking example of accommodation is given in (i). Although the uttered clauses provide no contrast, the speaker interprets the *séna7*-clause with a ‘not very well’ reading.

(i)

**Table:** 

*N-qwnúxw-alhts’a7*	***séna7***	*s-7ít’-em-s=a*	*s-Mary,*
loc-sick-inside	**cntr**	nmlz-sing-mid-3poss=exis	nmlz-Mary

*wá7=t’u7*	*t’it*	*n-qwnúxw-alhts’a7*	*snilh.*				
be=excl	also	loc-sick-inside	3sg.indep				
‘Mary’s song was sad, and she was also sad.’										
Consultant’s comment: “You’re saying Mary’s song is kind of sad – *séna7* is ‘kind of’.”

These show that *séna7* is unacceptable with states and activities if there is no salient proposition *q* which the speaker does not expect to be true at the same time as the prejacent.7Beside (32) and (36), additional monoclausal cases of *séna7* being rejected include (9) and (14) above. In these examples, the first clause was originally offered to the consultant and rejected. The sentences became fine when an appropriate *q* was added as follow-up.

(32)

**Table:** 

	Context: You went to see the Canucks. Qvlaotmec.wít iz’ kwa k’écwa7 (‘They’re bad at playing hockey’).	
#	*Ge7i7el’=wít=tu7*	***séna7***.
	lose=3pl=dist	**cntr**
	‘They lost.’ (cf. # They lost, all the same.)	
	Consultant’s comment: “I don’t think you really need *séna7* in there.”

(33)

**Table:** 

#	*Guy’t-ál’men=lhkan*	***séna7,***	*nilh*	*n=s=ka-gúy’t-a.*
	sleep-des=1sg.sbj	**cntr**	cop	1sg.poss=nmlz=circ-sleep-circ
	‘I was tired, so I fell asleep.’ (cf. # I was tired, but I still fell asleep.)

(34)

**Table:** 

Context: You and I and our sister Tina are supposed to be meeting at 7pm at the pizza place. It’s 7:15 and only you and I are there.							
Me:	*Nká7=tu7*	*s-Tina?*					
	where=dist	nmlz-Tina					
	‘Where’s Tina?’						
You: #	*O,*	*cuz’*	*áw’w’et*	*k=Tina,*	*wa7*	***séna7***	*guy’t-s-ás*
	oh	prosp	late	det=Tina	ipfv	**cntr**	sleep-caus-3erg
	i=stsmál’t-s=a.							
	pl.det=children-3poss=exis				
	‘Oh, Tina’s going to be late, she has to put her children to bed.’						
	(cf. # Oh, Tina’s going to be late, even though she has to put her children to bed.)

(35)

**Table:** 

*#*	*Lh=nás=acw*	***séna7***	*áku7*	*Calgary,*	*áma=ka*	*lh=sáq’w=acw.*
	comp=go=2sg.sbjv	**cntr**	deic	Calgary	good=deon	comp=fly=2sg.sbjv
	‘If you go to Calgary, you should fly.’					
	Consultant’s comment: “No, that *séna7* is not a good word in there.”					
	(cf. # Even if you go to Calgary, you should fly. (Note: Calgary is a long way away.))

(36)

A:

**Table:** 

*Kán-em=lhkacw*	*lhkúnsa?*
do.what-mid=2sg.sbj	now
‘What are you doing?’

B:

**Table:** 

#	*Wá7=lhkan*	***séna7***	*k’wezús-em.*
	ipfv=1sg.sbj	**cntr**	work-mid
	‘I’m working.’		
	Consultant’s comment: “Doesn’t make sense.” (cf. # I’m working, all the same.)

(37)

**Table:** 

*#*	*Gwel-en-ás*	***séna7***	*ta=np’ámsten=a,*	*nilh*	*s=púlh•elh=s*	*ta=qú7=a.*
	burn-dir-3erg	**cntr**	det=stove=exis	cop	nmlz=boil•fre=3poss	det=water=exis
	‘S/he lit the stove, and the water boiled.’ (cf. # S/he lit the stove, and the water still boiled.)					

In this section we have shown that *séna7* appears with states and activities when there is a failure of an expected outcome (including a failure of the eventuality to continue), or more generally when something unexpected happens during or after the eventuality, including cases where the activity is not performed successfully. If none of these conditions obtain (or can be reasonably accommodated), *séna7* is infelicitous.

### 2.2 *Séna7* with achievements and accomplishments

Achievement and accomplishment predicates behave similarly to each other in many respects when co-occurring with *séna7,* but there is one important difference relating to whether event culmination is entailed.8See Martin (2019), the papers in Martin and Demirdache (2020), and references therein, for discussion of non-culminating accomplishments across languages. For other Salish languages, see J. Davis (1978) and Watanabe (2003) on ʔayʔaǰuθəm (Comox-Sliammon), Bar-el (2005), Bar-el et al. (2005), and Jacobs (2011) on Skwxwú7mesh (Squamish), Gerdts (2008) on Hulq’umin’um (Island Halkomelem), and Kiyota (2008) and Turner (2011) on SENĆOŦEN (Northern Straits Salish). We will show that this difference provides support for our proposal that *séna7* cannot alter the truth conditions of its prejacent.

First, some background on these aspectual classes in St’át’imcets. Achievements are intransitive and unaccusative; they completely lack an external argument. Accomplishments are transitive and have agentive subjects. Crucially, achievements entail culmination in the perfective aspect, but accomplishments with control transitivizers do not: they merely implicate culmination (Bar-el et al. 2005; Matthewson 2004a).9A small class of transitive verbs formed from achievement roots do entail culmination. See below for the effect of *séna7* on this class. The basic facts are illustrated in (38)–(39). The same root, *mays* ‘get fixed’, entails culmination when it surfaces without transitivizing morphology (38), but only has a cancellable implicature of culmination when it appears with the directive (‘control’) transitivizer (39).10The implicature of culmination, along with other facts such as default tense interpretations and temporal behavior with punctual adverbials, are what distinguish accomplishments from activities in Salish languages. See for example Bar-el (2005), Kiyota (2008) for discussion.

(38)

**Table:** 

*#*	***Mays***	*ti=q’láxan=a,*	*t’u7*	*áoy=t’u7*	*kw=s=ka-máys=ts-a.*
	**get.fixed**	det=fence=exis	but	neg=excl	det=nmlz=circ-get.fixed=3poss-circ
	‘The fence got fixed, but it couldn’t be fixed.’				
	Consultant’s comment: “Contradiction.”				achievement

(39)

**Table:** 

*Máys-**en**=lhkan*	*ta=q’láxan=a,*	*t’u7*	*áoy=t’u7*
get.fixed**-** **dir** =1sg.sbj	det=fence=exis	but	neg=excl
*kw=s=ka-máys=ts-a.*				
det=nmlz=circ-get.fixed=3poss-circ						
‘I fixed a fence, but it couldn’t be fixed.’						accomplishment

When *séna7* is added to achievements and accomplishments, the former allow a subset of the interpretations allowed for the latter. The available interpretations are predictable given the difference between the two aspectual classes with respect to culmination entailments.

We begin with the ways in which achievements and accomplishments behave similarly, and then turn to the differences in the following sub-section.

#### 2.2.1 Similarities between achievements and accomplishments

*Séna7* can appear on achievements and accomplishments when an expected outcome of the event or of its result state fails to materialize. (40)–(43) show achievements, and (44)–(46) accomplishments.

(40)

**Table:** 

Context: I was invited to a meeting. I arrived there, and Lisa phoned and asked me if I got there. I reply:						
*Tsícw•ecw=kan*	***séna7,***	*t’u7*	*áy=t’u7*	*kwas*	*wá7*	*k=Laura.*
get.there•fre=1sg.sbj	**cntr**	but	neg=excl	det+nmlz+ipfv+3poss	be	det=Laura
‘I got there, but Laura wasn’t there.’

(41)

**Table:** 

*Tsícw=kan=t’u7*	***séna7** …*	*t’u7*	*xwem-7úl*	*kw=s=tsem’p=s,*
get.there=1sg.sbj=excl	**cntr**	but	quick-too	det=nmlz=finish=3poss

*nílh=t’u7*	*múta7*	*n=s=7úxwal’.*		
cop=excl	again	1sg.poss=nmlz=go.home		
‘I got there … but it was over already, so I came home.’

(42)

**Table:** 

*Ts’ék=tu7^11^*	***séna7***	*nelh=meláomen-s=a,*	*t’u7*	*plán=t’u7*	*wa7*
all.gone=dist	**cntr**	abs.pl.det=medicine-3poss=exis	but	already=excl	ipfv
*ama-wíl’c.*					
good-become					
‘His/her medicine was all gone, but s/he got better.’					

11 Although the predicate *ts’ek* ‘all gone’ translates into English as stative-like, it patterns as an achievement in St’át’imcets according to language-internal diagnostics (for example, behavior with the imperfective auxiliary *wa7* and with a ‘How long has …?’ construction).

(43)

**Table:** 

*Pún=lhkan^12^*	***séna7***	*i=n-neklí=ha,*	*t’u7…*	*plán=tu7*	*wa7*
find+dir=1sg.sbj	**cntr**	pl.det=1sg.poss-key=exis	but	already=dist	ipfv

*nak’*	*ta=xétsem-s=a*	*ta=neklí=ha.*						
change	det=box-3poss=exis	det=key=exis			
‘I found my keys … but the lock box has been changed.’					
*p:* *I found my keys*			*q:* *I can’t open the box*		

12 The transitive verb *pun* ‘find’ patterns as an achievement in St’át’imcets (enforcing culmination), even though it contains the directive transitivizer. It is part of a small class of transitive verbs that denote events whose running time is too short to allow initiation without culmination; since accomplishments in St’át’imcets require at least a portion of the event to take place, this results in achievement-like behavior.

(44)

**Table:** 

Context: Jim broke the neighbor’s fence by mistake.					
*Máys-en-as*	***séna7***	*ta=q’laxan-í=ha,*	*t’u7*	*wá7=t’u7*	*qlíl-min’-em.*
get.fixed-dir-3erg	**cntr**	det=fence-1pl.poss=exis	but	ipfv=excl	angry-rlt-pass
‘He fixed their fence, but they were mad at him anyway.’

(45)

**Table:** 

*Q’ets’-en=lhkán*	***séna7***	*ta=tsespíts’7=a,*	*t’u7*	*wá7=lhkan=t’u7*	*múta7*
knit-dir=1sg.sbj	**cntr**	det=sweater=exis	but	ipfv=1sg.sbj=excl	again
*es=yáon.*						
have=yarn						
‘I knitted a sweater, but I still have some yarn.’

(46)

**Table:** 

*Q’ets-cít=kan*	***séna7***	*ta=tsespíts’7=a*	*ta=n-kéckec=a,*	*t’u7*
knit-ind=1sg.sbj	**cntr**	det=sweater=exis	det=1sg.poss-older.sister=exis	but
*cw7áoy=t’u7*	*kwas*		*s-lhecw-s-ás.*	
neg=excl	det+nmlz+ipfv+3poss		stat-put.on-caus-3erg	
‘I made a sweater for my sister, but she didn’t wear it.’				

The second environment where *séna7* appears with achievements and accomplishments is when the expected result state of the event doesn’t hold. This is shown in (47)–(49) for achievements and in (50) for accomplishments; the expected result states are him being there, the fish being all gone, and ‘it’ being in a fixed state.

(47)

**Table:** 

*T’íq=k’a*	*séna7,*	*t’u7*	*cw7aoz*	*kwas*	*wa7*	*lhkúnsa.*
get.here=epis	**cntr**	but	neg	det+nmlz+ipfv+3poss	be	now
‘He must have arrived, but he’s not there now.’

(48)

**Table:** 

*Ts’áqw=t’u7*	***séna7***	*ti=sts’úqwaz’=a …*	*t’u7*	*cw7ít=t’u7*	*i=wá7*	*s-k’wilh.*
get.eaten=excl	**cntr**	det=fish=exis	but	many=excl	pl.det=ipfv	stat-left
‘The fish got eaten … but there were lots of leftovers.’

(49)

**Table:** 

*Máys=t’u7*	***séna7***	*inátcwas, …*	*t’u7*	*plan*	*múta7*	*qvl-wíil’c.*
get.fixed=excl	cntr	yesterday	but	already	again	bad-become
‘It got fixed yesterday … but it’s already broken again.’

(50)

**Table:** 

*Mays-en=lhkán=t’u7*	***séna7***	*inátcwas,*	*t’u7*	*plan*	*múta7*	*qvl-wíil’c.*
get.fixed-dir=1sg.sbj=excl	**cntr**	yesterday	but	already	again	bad-become
‘I fixed it yesterday, but it already broke again.’						

With both achievements (51)–(53) and accomplishments (54), *séna7* also allows an interpretation that the event didn’t happen well or successfully. (Notice that (51)–(52) contain the same predicate *mays* ‘get fixed’ as in (49), with a different interpretation.)

(51)

**Table:** 

*Máys=t’u7*	*séna7*	*ti=q’láxan=a …*	*t’u7*	*áoz=t’u7*	*kwas*
get.fixed=excl	**cntr**	det=fence=exis	but	neg=excl	det+nmlz+ipfv+3poss

*áma*	*kw=s=xilh-ts-twítas.*				
good	det=nmlz=do-caus-3pl.erg								
‘The fence got fixed … but they didn’t do it well.’

(52)

**Table:** 

*Máys=t’u7*	***séna7***	*ta=q’láxan=a,*	*t’u7*	*cw7áoz=t’u7*
get.fixed=excl	**cntr**	det=fence=exis	but	neg=excl

*kw=s=7i7éz’=s*	*kw=s=ca7=s,*	*nilh*		
det=nmlz=enough=3poss	det=nmlz=high=3poss	cop		

*s=lhegw-ilc-mín-itas*	*i=ts’í7=a.*
nmlz=jump-aut-rlt-3pl.erg	pl.det=deer=exis
‘The fence got fixed, but it wasn’t high enough, so the deer jumped over it.’

(53)

**Table:** 

*Nq’íxts=t’u7*	***séna7***	*ti=nk’wanústen=a,*	*t’u7*	*áy=t’u7*	*kwas*
closed=excl	**cntr**	det=window=exis	but	neg=excl	det+nmlz+ipfv+3poss
*stexw*	*ka-q’íxts-a.*								
really	circ-close-circ								
‘The window was closed, but it wasn’t closed properly.’

(54)

**Table:** 

*May-en-ítas=t’u7*	***séna7***	*ti=q’láxan=a …*	*t’u7*	*áoz=t’u7*
fix-dir-3pl.erg=excl	**cntr**	det=fence=exis	but	neg=excl

*kwas*	*áma*	*kw=s=xilh-twítas.*				
det+nmlz+ipfv+3poss	good	det=nmlz=do[-caus]-3pl.erg				
‘They fixed the fence, but they didn’t fix it well enough.’				

#### 2.2.2 Differences between achievements and accomplishments

Control accomplishments with *séna7* allow an interpretation which achievements do not allow: that the expected culmination of the event did not take place. This is illustrated in (55)–(58).

(55)

**Table:** 

*Mays-en=lhkán=t’u7*	***séna7***	*ti=q’láxan=a …*	*t’u7*	*áoy=t’u7*
get.fixed-dir=1sg.sbj=excl	**cntr**	det=fence=exis	but	neg=excl
*kw=s=tsúkw-s-an.*								
det=nmlz=finish-caus-1sg.erg								
‘I fixed the fence, but I didn’t finish.’

(56)

**Table:** 

*Mets-en=lhkán*	***séna7***	*ta=xzúm=a*	*nqwal’útten*	*pukw –*	*wá7=lhkan=t’u7*
write-dir=1sg.sbj	**cntr**	det=big=exis	language	book	ipfv=1sg.sbj=excl
*méts-en,*	*[t=]s=cw7áoy=s=a*			*kwenswá*				
write-dir	[det=]nmlz=neg=3poss=exis			det+1sg.poss+nmlz+ipfv				
*ka-tsúkw-s-a.*					
circ-finish-caus-circ										
‘I tried/am trying to write a dictionary, and I’m still writing it, because I can’t finish it.’

(57)

**Table:** 

*Tseg-ánk-en=lhkan*	***séna7***	*ta=ts’í7=a,*	*t’u7*	*ka-lhéxw-a*
tear-gut-dir=1sg.sbj	**cntr**	det=deer=exis	but	circ-appear-circ
*ta=st’alhálam=a*	*nilh*	*n=s=cúlel.*								
det=grizzly=exis	cop	1sg.poss=nmlz=run.away								
‘I was gutting a deer but a grizzly showed up and I ran away.’

(58)

**Table:** 

*Utsz-ay’lup-en-ítas*	***séna7***	*i=nguy’tten-í=ha*		
get.straight-bed-dir-3pl.erg	**cntr**	pl.det=bed-3pl.poss=exis		

*i=sk’wemk’úk’wmi7t=a,*	*t’u7 zaw’t-mín-itas,*	*nilh*	*s=tsicw=s*
pl.det=children=exis	but bored-rlt-3pl.erg	cop	nmlz=get.there=3poss

*sáy’sez’=wit*	*láku7*	*álts’q7=a.*	*Cw7áoy=t’u7*	*kw=s=7útsez*
play=3pl	deic	outside=exis	neg=excl	det=nmlz=get.straight

*i=ngúy’tten=a!*		
pl.det=bed=exis		
‘The children tried to fix their beds, but they got bored with it and went out to play. The beds weren’t fixed!’					

Crucially, achievements *cannot* fail to culminate with *séna7*. The consultant for (59) corrected the predicate to the accomplishment verb *máysen*, and in (60) the predicate was corrected to *zúqwalmen* ‘almost die’.

(59)

**Table:** 

#	*Máys=t’u7*	***séna7***	*ti=q’láxan=a,*	*t’u7*	*áoy=t’u7*
	fix=1sg.sbj=excl	**cntr**	det=fence=exis	but	neg=excl
	*kw=s=tsúkw-s-an.*							
	det=nmlz=finish-caus-1sg.erg							
	‘The fence got fixed, but I didn’t finish it.’

(60)

**Table:** 

#	*Zuqw*	***séna7***	*kw=s-Fred,*	*t’u7*	*ama-wíl’c*	*aylh.*
	die	**cntr**	det=nmlz-Fred	but	good-become	now
	‘Fred died, but he’s ok now.’									
	Consultant’s comment: “He died, but he’s still alive!”

(61)

**Table:** 

#	*Xelq’*	*ta=pal7-ál’ts=a*	*xzum*	*k’ét’a*	*lhl=áku7*	*cá7=a.*	*Qam’t*	***séna7***
	roll	det=one-rock=exis	big	rock	from=deic	high=exis	get.hit	**cntr**
	*kw=s-Bill*		*é=ta=k’ét’h=a,*		*t’u7*	*cík’-en-em.*					
	det=nmlz-Bill		prep=det=rock=exis		but	miss-dir-pass	
	‘A big rock rolled down from up high. Bill got hit, but he got missed.’							
	Consultant’s comment: “It’s conflicting because he got hit and missed.”							

The fact that achievements can never fail to culminate in the perfective aspect with *séna7* is an important finding: it shows that while *séna7* encodes an unexpected outcome or occurrence, it cannot take away entailments. *Séna7* does not alter the truth conditions of the proposition to which it attaches. This means that *séna7* cannot be captured by the analysis proposed for the Kimaragang frustrative by Kroeger (2017); we return to this point in Section 5.

The proposal that *séna7* cannot cancel the truth conditions of its prejacent correctly predicts that even with accomplishments, some part of the event, specifically its initial process part, still has to happen. Thus, *séna7* does not license an interpretation in which the event fails to start at all. This is shown in the minimal triplet in (62)–(64): two different unexpected occurrences are possible in (62)–(63), but it is not possible for no cooking at all to happen, as in (64).

(62)

**Table:** 

*Q’wel-en=lhkán*	***séna7***	*ta=sts’úqwaz’=a,*	*t’u7*	*cw7aoy=s*
cook-dir=1sg.sbj	**cntr**	det=fish=exis	but	neg=3poss
*kw=s=put=s*		*q’wel.*			
det=nmlz=exactly=3poss		cooked			
‘I cooked the fish, but it wasn’t cooked enough.’

(63)

**Table:** 

*Q’wel-en=lhkán*	***séna7***	*ta=sts’úqwaz’=a,*	*t’u7*	*cw7áoy=t’u7*
cook-dir=1sg.sbj	**cntr**	det=fish=exis	but	neg=excl
*ku=ts’aqw-an’-táli.*				
det=eat-dir-nts				
‘I cooked the fish, but nobody ate it.’

(64)

**Table:** 

#	*Q’wel-en=hlkán*	***séna7***	*ta=sts’úqwaz’a,*	*t’u7*	*cw7aoz*
	cook-dir=1sg.sbj	**cntr**	det=fish=exis	but	neg
	*kw=s=ka-gwél-s-an-a*			*ta=np’ámsten=a.*				
	det=nmlz=circ-burn-caus-1sg.erg-circ			det=stove=exis				
	‘I cooked the fish, but I wasn’t able to light the stove.’				

We also correctly predict that a set of transitive predicates which truth-conditionally *do* entail culmination in the perfective aspect (a subset of predicates formed from achievement roots and containing the *causative* transitivizer) still must culminate with *séna7;* this is shown in (65).

(65)

**Table:** 

#	*Nlíg’wts-**s**-as*	***séna7***	*ta=sk’éxem=a*	*ta=séps=a,*	*t’u7*	*wá7=t’u7*
	open- **caus** -3erg	**cntr**	det=wind=exis	det=door=exis	but	ipfv=excl
	*nq’ixts.*					
	closed					
	‘The wind opened the door but it’s still closed.’						
	Consultant’s comment: “It’s open and closed.”^13^						

13 Transitive verbs with inanimate subjects always culminate and obligatorily take the causative transitivizer *-s* rather than the directive (control) transitivizer *-Vn*.

*Séna7* with these culminating predicates gives rise to the usual unexpected/unsuccessful interpretations, as for example in (66)–(67).

(66)

**Table:** 

Context: You catch a kid breaking your window.				
*Lepinitás**-ts**=kan*	***séna7**,*	*t’u7*	*múzmit-s=kan*	*aylh.*
punish-**caus** =1sg.sbj	**cntr**	but	pity-caus=1sg.sbj	then
‘I punished him, but I took pity on him (I didn’t punish him hard).’

(67)

**Table:** 

*Sek’w-p-**s**=kán*	***séna7***	*na=nk’wnústen=a,*	*t’u7*	*áoy=t’u7*
break-inch-**caus** =1sg.sbj	**cntr**	abs.det=window=exis	but	neg=excl
*kw=n=s=ka-7úts’q7-a.*								
det=1sg.poss=nmlz=circ-go.out-circ								
‘I broke the window, but I couldn’t get out.’								
Consultant’s comment: “A window outside and a bar inside, that’s why he couldn’t get out.”				

### 2.3 Summary of empirical landscape

Table 1 summarizes the interpretations we have discovered with *séna7* for each Aktionsart. The result state and culmination tests are not applicable to states or activities, since these do not involve changes into result states.14We have not systematically tested the small class of semelfactives (telic, punctual events without result states), but (i) is an example:

(i)

**Table:** 

*Wa7*	***séna7***	*pegw-ts-ám’*	*k=Henry*	*kéla7*	*lhel=kw=s=7úlhcw=s,*
ipfv	**cntr**	knock-mouth-mid	det=Henry	first	prep=det=nmlz=enter=3poss
*t’u7*	*áy=t’u7*	*kw=s=qan’ím-ens-tum.*				
but	neg=excl	det=nmlz=hear-dir-1pl.erg			
‘Henry did knock before coming in, but we didn’t hear him.’

**Table 1:** Interpretations with *séna7*.

	Unexpected outcome/co-occurring event	Unsuccessful event	Failure of result state	Failure of culmination
States	√	√	N/A	N/A
Activities	√	√	N/A	N/A
Achievements	√	√	√	*
Accomplishments	√	√	√	√

We propose that all the attested semantic effects can be unified under a single generalization: *séna7* marks the unexpected co-occurrence of two true propositions. The columns in the table are thus not separate readings, but simply common ways in which the conditions on *séna7* can be met. In many cases, the proposition *q* which contrasts with the prejacent is provided by a generalized implicature deriving from the lexical semantics of the prejacent’s predicate: the implicatures that accomplishments will culminate, that achievements and accomplishments have persistent result states, and that eventualities will happen successfully.

The explanation for the lack of a ‘failure of culmination’ interpretation with achievements is, as already discussed, that *séna7* does not have the power to defeat entailments of the proposition to which it applies.

In the next section we formalize our analysis further, and go through some finer predictions it makes.

## 3 Analysis and detailed predictions

Our proposal – that *séna7 (p)* conveys that speaker did not expect *p* to be true as well as another contextually salient proposition *q* – is stated more formally in (68).

(68)

**Table:** 

[[ *séna7 (p)* ]]^c,w^ =	
At-issue:	[[ *p* ]]^c,w^
Not-at-issue:	∃q [(q(w) = 1) & ¬∃w’ [w’ ∈ BEST_STEREO(w)_(∩EPIS_sp(c)_(w)): p(w’) = 1 & q(w’) = 1]]

In this formula, EPIS_sp(c)_ is an epistemic modal base for a speaker in a context *c*. ∩EPIS _sp(c)_(w) is the set of worlds which are epistemically accessible to the speaker of *c* in *w* (worlds which are compatible with the speaker’s beliefs)*.* STEREO is an ordering source; BEST_STEREO(w)_ orders a set of worlds according to stereotypicality relative to *w,* and selects the most stereotypical ones.

Putting all this together, the speaker of *séna7(p)* asserts *p,* and conveys at a not-at-issue level that there is a true proposition *q,* and there is no world w’ among the most stereotypical worlds epistemically accessible to the speaker such that *p* and *q* are both true in *w’.*

This is a modal analysis, using standard Kratzerian conversational backgrounds (an epistemic modal base, a stereotypical ordering source). There is thus a similarity with familiar epistemic modals, as in (69).

(69)

**Table:** 

*Michl **must** be the murderer.*
(Kratzer 1991: 643)

A standard analysis of (69) is that it means ‘In all worlds which are compatible with the speaker’s beliefs/evidence (epistemic modal base), and in which things proceed in a maximally normal manner (stereotypical ordering source), Michl is the murderer.’ In our analysis, *séna7* quantifies over the same set of worlds as an epistemic modal, but conveys that there are *no* maximally stereotypical epistemically accessible worlds in which *p* and *q* are both true. Another important difference between ordinary epistemic modals like *must* and *séna7* is that *séna7* conveys modality in the not-at-issue realm: it doesn’t directly assert its modal contribution.

Our analysis makes some further detailed predictions. These are laid out in (70) and tested in the following sub-sections.

(70)

**Table:** 

Predictions of the analysis	
i.	*Séna7* takes only one syntactic argument (its prejacent clause).
ii.	The unexpectedness requirement is symmetrical between the two propositions.
iii.	*Séna7*’s contribution cannot scope under other operators.
iv.	The unexpectedness requirement holds only for the speaker.
v.	*Séna7*’s prejacent clause need not be inherently unexpected.
vi.	The requirement is about expectations, not intentions.
vii.	The requirement is about expectations, not causality.

### 3.1 *Séna7* takes only one syntactic argument

*Séna7* applies to a prejacent proposition *p*; however, the contrasting proposition *q* is not an argument of *séna7,* but is existentially quantified over. This predicts that *q* is not syntactically required to be present. A strong piece of evidence for this is that mono-clausal sentences containing *séna7* are possible (and frequently volunteered). We have seen several examples of this above, and (71)–(73) are more cases where the contrasting proposition *q* is not overtly given.

(71)

**Table:** 

Context: Seven people are trying to get into a car. The driver says:			
*Xzum*	***séna7***	*ti=n-káoh=a.*	
big	**cntr**	det=1sg.poss-car=exis	
‘My car is big.’			
Consultant’s comment: “Means they can’t all fit in.”			
*p:* *My car is big*		*q:* *They can’t all fit in*

(72)

**Table:** 

Context: I got burned when I was a child. My mother was working out there in the back. … My brother Dicky was around. He was helping my mother there. So my mother told him, “Go look at the baby, and see if she’s okay.” So he went inside.					
*Tsicw,*	*s=7áts’x-en-as*	*láti7*	***séna7***	*s-law*	*l=ti=tsepalín=a.*
get.there	nmlz=see-dir-3erg	deic	**cntr**	stat-hang	prep=det=baby.basket=exis
‘He got there and saw that she (the baby) was hanging in the basket, sure enough.’					
(Laura Thevarge, in Matthewson 2005: 272–273)					
*p:* *The baby was hanging in the basket*				*q:* *The baby wasn’t alright*

(73)

**Table:** 

Context: I was sick yesterday.			
*Xát’-min’=lhkan*	***séna7***	*kw=n=s=tsunám’-cal.*	
want-rlt=1sg.sbj	**cntr**	det=1sg.poss=nmlz=teach-act	
‘I wanted to teach.’			
*p:* *I wanted to teach*		*q:* *I didn’t teach*	

Even when there are two overt clauses, the contrasting proposition *q* is not necessarily represented by one of them. In (74), for example, it is not unexpected that a potential place to stay would be both good and expensive, so the contrast is not between the two overt clauses. Rather, the fact that the place seems good *(p)* contrasts with the implicitly conveyed proposition *q* ‘We won’t stay here’.

(74)

**Table:** 

Context: A asks B ‘Shall we stay here?’ B replies:					
*Áma=t’u7*	*lákw7a*	***séna7,***	*t’u7*	*kéla7=t’u7*	*cw7it-usa7-[7]úl.*
good=excl	deic	**cntr**	but	very=excl	much-money-too
‘It seems good, but it is very expensive.’					
*p:* *It seems good*			*q:* *We won’t stay here*				

In (75), *séna7* encodes the unexpectedness of my not having another drink, even though I have money. Crucially, *q* is not the second overt clause, ‘I’ve already had enough to drink’. Instead, *q* is an implicature of the second overt clause.

(75)

A:

**Table:** 

*Cúz’=lhkacw=ha*	*úqwa7*	*ku=pála7*	*múta7?*
prosp=2sg.sbj=q	drink	det=one	more
‘Are you going to have another drink?’

B:

**Table:** 

*Cw7ao.*
neg
‘No.’

A:

**Table:** 

*Icwa7=lhkácw=ha*	*es=qláw’?*
without=2sg.sbj=q	have=money
‘Don’t you have any money?’

B:

**Table:** 

*Wá7=lhkan*	***séna7***	*es=qláw’,*	*t’u7*	*plan*	*í7ez’*
ipfv=1sg.sbj	**cntr**	have=money	but	already	enough

*n-s-7úqwa7.*					
1sg.poss-nmlz-drink					
‘I have money, but I’ve already had enough to drink.’						
*p:* *I have money*		*q:* *I’m not having another drink*

Similarly in (76), *q* is provided by conversational implicature. Here, *séna7* is contrasting going out with not having fun, which is implicated by not having any money.

(76)

**Table:** 

*Saotatih-am=lhkán=tu7*	***séna7***	*inátcwas,*	*t’u7*	*ícwa7=lhkan*	
Saturday-mid=1sg.sbj=dist	**cntr**	yesterday	but	without=1sg.sbj	
*es=qláw’.*					
have=money					
‘I went out yesterday, but I didn’t have any money.’					
Consultant’s comment: “He went, but he didn’t have any money so he didn’t have much fun.”					
*p:* *I went out*	*q:* *I didn’t have much fun*				

### 3.2 The unexpectedness requirement is symmetrical between the two propositions

According to our proposal, the unexpectedness requirement of *séna7* targets two propositions (*p* and *q*) symmetrically. That is, although *séna7* syntactically appears within one clause (the *p* clause), the not-at-issue relation it expresses does not prioritize one proposition over the other. This is supported by the fact that in a linear sequence of two clauses, *séna7* is not restricted to appearing in the first one. Although it appears on the first clause in most of our data, there are second-clause examples, as shown in (77)–(80). Example (78) is a minimal pair with (21) and is interpreted identically, showing that the clause *séna7* is placed in has no effect on the meaning; Examples (94)–(97) below provide two more minimal pairs with *séna7* in opposite clauses, with no effect on meaning.15There can even marginally be two *séna7*’s in one sentence, as in (i).

(i)

**Table:** 

*N-qwnúxw-alhts’a7*	***séna7***	*ta=s-7ít’-em-s=a*	*s-Mary,*	*t’u7*	*áma*
loc-sick-inside	**cntr**	det=nmlz-sing-mid-3poss=exis	nmlz-Mary	but	good
***séna7***	*ta=scwákwekw-s=a.*					
**cntr**	det=heart-3poss=exis					
‘Mary sang a sad song, even though she was happy.’					
Consultant’s comment: “I guess I’d let it pass.”

(77)

**Table:** 

*ma* *ku=syáqtsa7,*	*t’u7*	*cw7aoz*	***séna7***	*kwas*
good det=woman	but	neg	**cntr**	det+nmlz+ipfv+3poss
*s-lhík-s-as*		*ku=wá7* *kukw.*		
stat-clear-caus-3erg		det=ipfv cook		
‘There’s a nice lady out there, but she doesn’t know how to cook.’

(78)

**Table:** 

*N-qwnúxw-alhts’a7*	*[ta=]s-7ít’-em-s=a*	*s-Mary,*	*t’u7*	*áma*	***séna7***
loc-sick-inside	[det=]nmlz-sing-mid-3poss=exis	nmlz-Mary	but	good	**cntr**
*ta=scwákwekw-s=a.*										
det=heart-3poss=exis										
‘Mary’s song/singing was sad, but she is happy.’

(79)

**Table:** 

*N-wá7-ten-s*	*ku=ts’í7*	*na=pún-an=a*					
loc-be-ins-3poss	det=deer	abs.det=find+dir-1sg.erg=exis					
*i=wan*		*píxem’,*	*aoz*	***séna7***	*ku=ts’í7.*
when.pst=ipfv+1sg.sbjv		hunt	neg	**cntr**	det=deer
‘I found a deer’s bedding place when I went hunting, but there weren’t any deer.’					
(Alexander et al. in prep.)

(80)

**Table:** 

*Nzah-en-tsálem*	*aylh,*	*wá7=lhkan*	***séna7***	*kens-téxw-en*	*i=tewtwéw’wet=a.*
right-dir-1sg.pass	then	ipfv=1sg.sbj	**cntr**	try-straight-dir	pl.det=boys=exis
‘The boys got the better of me when I was trying to correct them.’					
(Alexander et al. in prep.)					

### 3.3 *Séna7*’s contribution cannot scope under other operators

Our claim that *séna7* contributes its semantic content in the not-at-issue dimension predicts that its unexpectedness contribution cannot take scope under operators such as negation. This is correct. In (81), the negation targets the at-issue truth conditions of the first clause (they did *not* allow us to run and play); it crucially does not negate the unexpectedness (i.e., the sentence does not mean ‘It is not unexpected that we ran and played in spite of them not allowing us to’).

(81)

**Table:** 

***Áoz**=k’a*	***séna7***	*kwas*	*cw7an-tumúlh-as*	
**neg**=epis	**cntr**	det+nmlz+ipfv+3poss	allow+dir-1pl.obj-3erg	

*kwetwá*		*wa7*	*q’í•q’•lhil*	*kenáti7*	*sáy’sez’.*
det+nmlz+ipfv+1pl.subj		ipfv	run•cre•	around	play
‘They didn’t allow us to run around playing.’					
(Gertrude Ned, in Matthewson 2005: 202)					
*p:* *They didn’t allow us to run and play*			*q:* *We ran and played*			

Similarly in (82), *séna7*’s contribution is not targeted by the negation. The sentence asserts that the speaker didn’t want to go to the school, and *séna7* contrasts the lack of wanting to go with the fact that he had to go anyway.

(82)

**Table:** 

*Cw7aoz*	***séna7***	*kw=n=s=xát’-min’*	*kw=n=s=nas*
neg	**cntr**	det=1sg.poss=nmlz=want-rlt	det=1sg.poss=nmlz=go
*ta=tsunám’-cal-ten=a.*						
[prep=]det=teach-act-ins=exis						
‘I didn’t want to go to the school.’				
(adapted from Carl Alexander, in Alexander 2016: 173)				
*p:* *I didn’t want to go to the school.’*		*q:* *I went to school*	

### 3.4 The unexpectedness requirement holds only for the speaker

The requirement that *p* and *q* are not expected to both be true is placed only on the speaker. This predicts that the addressee need not share the speaker’s assumptions about what counts as unexpected. We test this in (83)–(84). Here, the contexts do not provide a contrasting *q* for the addressee, yet the sentences are fine.16This makes *séna7* different from Zeevat’s (2005) adversative markers, which he analyzes as relying on what the common ground entails.

(83)

**Table:** 

Context: I never thought that my friend would win the race, but she always thinks she’ll come in first. The day of the race comes, and she wins by miles! I say to her:			
*T’cúm=lhkacw*	***séna7**!*		
win+mid=2sg.sbj	**cntr**		
‘You won anyway!’			
*p:* *You won*		*q:* *You aren’t a good enough runner to win*

(84)

**Table:** 

Context: Your friend and you have different ideas of what counts as a fun activity and you often disagree about it. The friend thinks that the best thing is to go to a large gathering and sing and dance. You much prefer to stay home and be quiet with the family. Yesterday, you went to a large gathering. Today you tell your friend:					
*Tsícw=kan*	***séna7***	*áta7*	*xzúm=a*	*s-gaw’p,*	*qwámqwmet-s=kan!*
get.there=1sg.sbj	**cntr**	deic	big=exis	nmlz-gather	fun-caus=1sg.sbj
‘I went to a big gathering, I had fun!’					

### 3.5 *Séna7*’s prejacent clause need not be inherently unexpected

According to our analysis, the speaker of a *séna7*-clause does not expect the prejacent to be true at the same time as some other salient proposition *q.* The speaker crucially does not have to believe that the prejacent proposition itself is inherently unexpected. We see this in (85)–(86); in these cases, the prejacent of *séna7* when considered in isolation is stereotypically often true.

(85)

**Table:** 

*Ka-cát-q-a*	***séna7***	*ta=snéqwem=a,*	*t’u7 …*	*qwelqúl’,*	*nilh*
circ-rise-bottom-circ	**cntr**	det=sun=exis	but	cloudy	cop
*s=cw7aoy=s*		*kw=s=7áts’x-en-em.*							
nmlz=neg=3poss		det=nmlz=see-dir-1pl.sbj							
‘The sun came up … but it was cloudy, so we couldn’t see it.’

(86)

**Table:** 

*Saq’w*	***séna7***	*i=spepzúz7=a,*	*t’u7 …*	*cw7aoz*	*kw=s=ca7=s.*
fly	**cntr**	pl.det=birds=exis	but	neg	det=nmlz=high=3poss
‘The birds flew … but not high.’					

### 3.6 The requirement is about expectations, not intentions

The unexpected co-occurrence of *p* and *q* includes, but is not limited to, situations where some agent had an intention which failed. In (12) above, *séna7* accompanies a report of a failed plan (to kill deer), but in (87), there was no plan that ‘they’ (riders in a ‘suicide race’) would get hurt. The speaker simply did not expect them to escape unscathed from this dangerous situation.

(87)

**Table:** 

*K’ínk’net=ti7*	***séna7,***	*t’u7*	*cw7aoz*	*kw=s=wá7=wit*	*xan’.*
dangerous=dem	**cntr**	but	neg	det=nmlz=ipfv=3pl	get.hurt
‘It was dangerous, but they didn’t seem to get hurt.’					
(Beverley Frank, in Matthewson 2005: 92)					

Further cases where there is no failed intentional plan are given in (88)–(89).

(88)

**Table:** 

*Kwís=tu7*	***séna7***	*n-káoh=a*	*lhél=ta=c.wálh=a,*		*t’u7*
fall=dist	**cntr**	1sg.poss=car=exis	prep=det=road=exis		but
*ken’•n’-alqw-mín-as*			*láti7*	*ta=xzúm-al’ts=a*	*k’ét’a,*	*nilh*
bump•fre-log-rlt-3erg			deic	det=big-rock=exis	rock	cop
*s=ka-t’ál=s-a.*								
nmlz=circ-stop=3poss-circ								
‘The car rolled off the road, but it hit a rock, and that stopped it.’					
*p:* *The car rolled off the road*			*q:* *The car did not continue to roll*

(89)

**Table:** 

*Ka-gwél-s-as-a*	***séna7***	*ta=nléqemten=a*	*i=sxéz’p-s=a*
circ-burn-caus-3erg-circ	**cntr**	det=hayfield=exis	pl.det=spark-3poss=exis
*ta=sp’áms-kalh=a*	*láti7,*	*t’u7 ka-lhap-s-tum’-á=hem’=tu7.*	
det=fire-1pl.poss=exis	deic	but circ-put.out-caus-1pl.erg-circ=anti=dist	
‘The hayfield caught fire from the sparks of our fire, but we got it out.’			
*p:* *The hayfield caught fire*		*q:* *The hayfield did not continue to burn*	

These data show that the contribution of *séna7* cannot be unified in terms of involving frustrated intention. However, the data *can* all be unified in terms of unexpectedness. Our proposal that St’át’imcets *séna7* does not semantically convey frustrated intention is in accord with Overall’s (2017: 485) observation that in the Amazonian languages he discusses, “The sense of unfulfilled *intention* or *desire …* seems in most cases to be epiphenomenal.”

### 3.7 The requirement is about expectations, not causality

We gave examples above where the unexpectedness of *p* and *q* both being true did not derive from a failed causal relation ((21)–(22)); further examples are given here. In (90), the issue is not that their teaching us to cook *(p)* is expected to *cause* them to know how to cook (the negation of *q*). Rather, it is simply unexpected for *p* and *q* to both be true.17As a reviewer points out, in the second clause of (90) *séna7* could also be conveying a ‘not very well’ interpretation. Similarly in (91), the chicken being cooked *(p)* would not *cause* the potatoes to be cooked (the negation of *q*), in (92), the fence getting fixed would not *cause* the gate to be fixed, and in (93) getting to the meeting would not *cause* the car not to break down, yet *séna7* is fine in all three examples.

(90)

**Table:** 

*Aoz*	*n-scwákwekw*	*kwas*	*s-lhik-s-twítas*	*kwa*
neg	1sg.poss-heart	det+nmlz+ipfv+3poss	stat-clear-caus-3pl.erg	det+ipfv
*kukw i=núkw=a.*			*Wa7 tsunam’-en-túmulh-as **séna7.***		
cook pl.det=some=exis			ipfv teach-dir-1pl.obj-3erg **cntr**		
‘I think some of them didn’t know how to cook. But they taught us [to cook] anyway.’				
(Rose Whitley, in Matthewson 2005: 475–476)				
*p:* *They taught us to cook*			*q:* *They didn’t know how to cook*

(91)

**Table:** 

Context (translated from St’át’imcets): I cooked for my relatives. I thought that the potatoes and the chicken would be ready together.				
*Q’wel*	***séna7***	*ta=tsíken=a,*	*t’u7*	*cw7áoy=t’u7*
get.cooked	**cntr**	det=chicken=exis	but	neg=excl

*kw=s=q’wel=s*			*i=petáok=a.*				
det=nmlz=get.cooked=3poss			pl.det=potato=exis				
‘The chicken got cooked but the potatoes didn’t.’ (adapted from Alexander et al. in prep.)

(92)

**Table:** 

*Mays*	***séna7***	*ta=q’láxan=a,*	*t’u7*	*cw7áoz=t’u7*	*kw=s=mays=ts*
get.fixed	**cntr**	det=fence=exis	but	neg=excl	det=nmlz=get.fixed=3poss
*ta=nq’íxtsten=a.*										
det=gate=exis										
‘The fence got fixed but the gate didn’t.’

(93)

**Table:** 

*Qacw•cw-áwlh*	*t’u7*	*tsícw•ecw*	***séna7***	*l=ta=s-gáw’p=a.*
break•fre-vehicle	but	get.there•fre	**cntr**	prep=det=nmlz=gather=exis
‘His car broke down, but he made it to the meeting anyway.’				

In (94), *séna7* is licensed by the common expectation that of the spring salmon run at the same time as the strawberries are ripe. However, there is no causal connection between the salmon running and the berries ripening; it is simply that they ripen at the same time of year. As further evidence that causality is not involved here, we elicited this sentence also with *séna7* in the opposite clause, as shown in (95).

(94)

**Table:** 

*Plan*	***séna7***	*t’ak*	*i=zúmak=a,*		*t’u7*
already	**cntr**	go.along	det.pl=spring.salmon=exis		but

*cw7áoy=s=t’u7*	*kwas*			*q’wel*	*i=sq’wláp=a*					
[nmlz=]neg=3poss=excl	det+nmlz+ipfv+3poss			ripe	det.pl=strawberry=exis					
‘The spring salmon are already running, but the strawberries aren’t ripe yet.’

(95)

**Table:** 

*Plan*	*t’ak*	*i=zúmak=a,*	*t’u7*	*cw7áoy=s=t’u7*
already	go.along	det.pl=spring.salmon=exis	but	[nmlz=]neg=3poss=excl
***séna7***		*kwas*	*q’wel*	*i=sq’wláp=a.*	
**cntr**		det+nmlz+ipfv+3poss	ripe	det.pl=strawberry=exis	
‘The spring salmon are already running, but the strawberries aren’t ripe yet.’				
Consultant’s comment: “Ts’íla t’ú7 ti7 ta núkwa, áma.” (“Like the other one [(94)], good.”)				

Examples (96) and (97) are identical except that *séna7* appears in the first versus second clause. A causal analysis would have to conclude that the examples have quite different meanings, an idea for which there is no evidence. Moreover, each of the potential causal claims are somewhat implausible: either that liking to eat cake causes one to not be able to each much cake (96), or that not being able to eat much cake causes one to like eating cake (97).

(96)

**Table:** 

*Texw=kán=t’u7*	***séna7***	*wa7*	*áma-s*	*ku=ts’aqw-an’-táli*	*i=kíks=a,*
very=1sg.sbj=excl	**cntr**	ipfv	good-caus	det=eat-dir=nts	det.pl=cake=exis

*t’u7*	*wa7=lhkan=ká=t’u7*	*s-7ats’x-s*	*n-mezáts=a,*			
but	ipfv=1sg.sbj=irr=excl	stat-see-caus	[det=]1sg.poss-body=exis			
*ay=s*	*kw=en=xmank.*								
neg=nmlz	det=1sg.poss=[nmlz=]heavy								
‘I really like eating cake, even though I have to watch my weight.’					
(Lit.: ‘I really like eating cake, but I have to take care of my body so I don’t get heavy.’)					
*p:* *I really like eating cake*		*q:* *I can’t eat much cake*

(97)

**Table:** 

*Texw=kán=t’u7*	*wa7*	*áma-s*	*ku=ts’aqw-an’-táli*	*i=kíks=a,*	*t’u7*
very=1sg.sbj=excl	ipfv	good-caus	det=eat-dir=nts	det.pl=cake=exis	but
*wa7=lhkan=ká=t’u7*			***séna7***	*s-7ats’x-s*	*n-mezáts=a,*		
ipfv=1sg.sbj=irr=excl			**cntr**	stat-see-caus	[det=]1sg.poss-body=exis		
*ay=s*			*kw=en=xmank.*						
neg=nmlz			det=1sg.poss=[nmlz=]heavy						
‘I really like eating cake, even though I have to watch my weight.’					
*p:* *I can’t eat much cake*			*q:* *I really like eating cake*				

A final piece of evidence that the two propositions *séna7* relates need not stand in a causation relation is given in (98). In this context, the event of reaching Mount Currie is part of a larger event of traveling to Lillooet from Vancouver. Yet a causing and a caused event must be fully distinct and cannot stand in a part-whole relation (Menzies and Beebee 2020).

(98)

**Table:** 

Context: Susie lives in Vancouver and has relatives in both Mount Currie and Lillooet. When she gets time off work she likes to visit her relatives, but she only has time to visit one set per trip. So she either comes directly to Lillooet (in which case she doesn’t usually go through Mount Currie), or she visits relatives in Mount Currie and then goes straight back home, without going to Lillooet. However, this time she surprised us:				
*Tsicw*	***séna7***	*ta=líl’wat7úl=a,*	*nílh=t’u7*	*s=tsicw=s*
get.there	**cntr**	[prep=]det=Mount.Currie=exis	cop=excl	nmlz=get.there=3poss
*áta7*	*sát’=a.*									
deic	Lillooet=exis								
‘She went to Mount Currie, but then she went on to Lillooet.’				

This will be important below where we argue that Copley and Harley’s (2014) cause-based analysis of frustratives cannot be applied to St’át’imcets *séna7.*

Having shown that the fine-grained predictions of our analysis are upheld, we turn now to further implications. We show that besides the distinction between achievements and accomplishments discussed in Section 2.2, there are other subtle semantic distinctions in the language which *séna7* allows us to delineate clearly, further highlighting its use as a diagnostic tool for illuminating contrasts beween entailments and implicatures. In Section 4.1 we undertake an examination of future time reference, showing that *séna7* allows us to distinguish between a future modal and a prospective auxiliary, and in Section 4.2 we extend the diagnostic to show that some motion verbs must contain prospective semantics.

## 4 *Séna7* and future time reference

### 4.1 Future versus prospective aspect

When *séna7* co-occurs with markers of future time reference, the results are as predicted by the analysis. Furthermore, *séna7* distinguishes semantically between the two grammaticized forms of future time reference in St’át’imcets.

The two grammatical markers of future time reference in St’át’imcets are the modal clitic *=kelh* and the aspectual auxiliary *cuz’.* Both appear in (99). As a rough approximation*, =kelh* corresponds to English *will* or future-oriented *might,* while *cuz’* corresponds to *is going to.* See van Eijk (1997), Matthewson (2006b), Rullmann et al. (2008) and Davis (2017) for discussion.

(99)

**Table:** 

***Cúz’**=lhkalh*	*ncwíl-cal*	*ku=kosoh-álhts’a7.*	*Ncwil-in’-ém=**kelh***	
**prosp** =1sg.sbj	roast-act	det=pig-meat	roast-dir-1pl.erg= **fut**	
*ku=cín’.*				
det=long.time				
‘We’re going to roast some pork. We will roast it for a long time.’				
(Alexander et al. in prep)				

We assume a neo-Reichenbachian approach to tense and viewpoint aspect that involves reference to (at least) three time intervals: a reference time (the time about which the sentence makes a claim), an event time (the time at which the event takes place), and an evaluation time (with respect to which tenses are evaluated). The evaluation time is by default the utterance time in matrix contexts. Thus, for example, a past tense places the reference time before the utterance time in a matrix clause. See Klein (1994) for this type of approach.

Glougie (2008) argues that the St’át’imcets clitic *=kelh* places the reference time after the evaluation time, while *cuz’* is a prospective aspect which places the event time after the reference time.18Glougie also argues that *cuz’* differs from *=kelh* in not introducing modality; we remain agnostic about this here. The modality question is independent of what is crucially distinguished by *séna7,* which is the relation between utterance time, reference time, and event time. In simple cases, the meanings are difficult to tease apart, but Glougie shows that the elements diverge in cases where an event is already planned at the utterance time. Here, only *cuz’* is acceptable, not *=kelh,* as shown in (100). Glougie notes that (100b) would be appropriate if the speaker was only considering going away for the weekend and had not yet purchased a bus ticket.19Relatedly, the two futurity markers also diverge when it comes to offering contexts as discussed by Copley (2002, 2009: only *=kelh* can be used to make a felicitous offer, not *cuz’*.

(100)

**Table:** 

Context: You are going to D’Arcy for the weekend. You have already purchased your bus ticket, and you leave tomorrow morning at 8:00am. I ask you what your plans are for the weekend. How do you respond?

a.

**Table:** 

***Cúz**’=lhkan*	*nas*	*áku7*	*nk’.wátqwa*	*natcw.*
**prosp** =1sg.sbj	go.to	deic	D’Arcy	tomorrow
‘I am going to D’Arcy tomorrow.’

b.

**Table:** 

#	*Nás=kan=**kelh***	*áku7*	*nk’.wátqwa*	*natcw.*
	go.to=1sg.sbj=**fut**	deic	D’Arcy	tomorrow
	‘I might go to D’Arcy tomorrow.’				
(Glougie 2008)

With both *=kelh* and *cuz’,* the evaluation or reference time need not be the utterance time, but can be a past time. This is parallel to the situation in English, where *will* has a past-shifted form *would,* and *is going to* has a past-shifted form *was going to.* Past-shifted examples of *=kelh* and *cuz’* are given in (101) and (102) respectively.

(101)

**Table:** 

Context: Mike Leech is currently chief of T’ít’q’et. His (deceased) mother was called Julianne.			
*Zewát-en-as*	*s-Julianne*	*[kwas*	*kúkwpi7=**kelh***
know-dir-3erg	nmlz-Julianne	[det+nmlz+ipfv+3poss	chief= **fut**
*ta=skúza7-s=a]*		*i=kwís=as.*				
det=child-3poss=exis]		when.pst=fall=3sbjv			
‘Julianne knew when he was born that her child would become chief.’			
(Matthewson 2006b: 689)

(102)

**Table:** 

*Nás=kalh*	*áku7*	*ts’úqwaz’-am,*	*nilh*	*ti=s-tlh-áyen=a*	***cuz’***
go=1pl.sbj	deic	fish-mid	cop	det=nmlz-stretch-net=exis	**prosp**
*qwez-en-ém.*										
use-dir-1pl.erg					
‘We went fishing, we were going to use a gillnet.’					
(Beverley Frank, in Matthewson 2005: 54)					

When *séna7* co-occurs with these markers of future time reference, it gives rise to two quite distinct readings. With *=kelh, séna7* imparts that the event described by *p* will happen, in spite of some other proposition *q,* while with *cuz’, séna7* conveys that the prejacent event was going to happen, but the event described by *q* happened instead.

Data with *=kelh* are given in (103)–(104). Here, the speaker is making a prediction about a future event, and in addition there is some contextually recoverable true proposition *q,* and the speaker finds it unexpected that *q* is true as well as *p*.

(103)

**Table:** 

*Úqwa7=**kelh***	***séna7***	*ku=qú7.*	
drink= **fut**	**cntr**	det=water	
‘He will drink water.’		
Consultant’s volunteered context: If he was on a mountain, and he doesn’t know whether the water is good, but he’ll drink it anyway.			
*p: He will drink water*		*q: He doesn’t know if the water is good*

(104)

**Table:** 

*Ilhen=**kélh**=ti7*	***séna7**.*		
eat= **fut**=dem	**cntr**		
‘He’ll eat anyway.’				
Consultant’s volunteered context: When there’s a big line up, and they are running low on food, but they’ll serve him anyway.			
*p: He will eat*	*q: They are running low on food*

These data are as predicted by Glougie’s analysis of *=kelh* and ours of *séna7*. The future modal *=kelh* places the reference time after the evaluation time, which in these examples is the utterance time. *Séna7*’s prejacent, which contains *=kelh,* asserts that an event will take place at that future reference time in all relevant possible worlds. *Séna7* contributes that the speaker doesn’t expect that the future proposition *p* and some contextually available proposition *q* are both true. In other words, the speaker asserts that an event will happen in the future, and conveys that something unexpected will also happen. This gives an ‘in spite of’ or ‘anyway’ reading.

Data with *cuz’* are given in (105)–(108). Here we get a quite different interpretation.

(105)

**Table:** 

***Cúz’**=k’a*	*zam’*	***séna7***	*tsut*	*wa7*	*“qwa<7>ez’-álhmec”,*
**prosp** =epis	after.all	**cntr**	say	ipfv	blue<inc>belly
*nilh s=ka-tsút=s-a*					*“qwa<7>y-án’ak”=ku7.*
cop nmlz=circ-say=3poss-circ					blue<inc>belly=rep				
‘So he was apparently going to say he was ‘qwa7ez’álhmec’, but he accidentally said ‘qwa7yán’ak’ instead.’					
(Carl Alexander, in Alexander 2016: 190)

(106)

**Table:** 

*Nilh*	***séna7***	*n=s=**cuz’***	*p’án’t-s,*	*t’u7*	*ka-law-a=t’ú7=a*
cop	**cntr**	1sg.poss=nmlz= **prosp**	return-caus	but	circ-hang-circ=excl=a
*múta7.*
again
‘I tried to put it [my finger] back, but it was just hanging there.’						
(Carl Alexander, in Alexander 2016: 305)						
*p: I was going to put it back*			*q: I didn’t put it back*

(107)

**Table:** 

*Nílh=tu7*	***séna7***	*ku=s-Father Paterson*	* ku=**cúz’***
cop=dist	**cntr**	det=nmlz-Father.Paterson	kdet=**prosp**
*melyih-s-tumúlh-as,*		*t’u7*	*láni7=tu7*	*i=qwatsáts=as*	
marry-caus-1pl.obj-3erg		but	deic=dist	when.pst=leave=3sbjv	
*kn=[n]ká7=as*	*s-Father Paterson.*		
around=where=3sbjv	nmlz-Father.Paterson				
‘It was supposed to have been Father Paterson who was going to marry us, but Father Paterson had left and gone somewhere.’				
(Gertrude Ned, in Matthewson 2005: 213)

(108)

**Table:** 

***Cúz’**=lhkan*	***séna7***	*áz’-en*	*na=káoh=a,*	*t’u7*	*plán=tu7*	*wa7*
**prosp** =1pl.sbj	**cntr**	buy-dir	det=car=exis	but	already=dist	ipfv
*lhég•gep.*
*get.sold•fre*
‘I was going to buy the car, but it had already been sold.’						
(Alexander et al. in prep.)								

Again, the results fall out from the analysis. *Cuz’* places the event time after the reference time, which in these examples is a past time. *Séna7*’s prejacent thus makes a claim about a pre-state of an event (the state of something being about to happen). *Séna7* conveys that there is some other proposition *q* that is unexpected given the prospective *p* (the claim that there was a pre-state of an eventuality). The most natural case is that *q* entails that the expected eventuality did not take place. The *cuz’* data are similar to cases where *séna7*’s prejacent is a lexical stative, as discussed in Section 2.1. Just as *séna7* when applied to a proposition about wanting something frequently conveys that the expected outcome of that desire (getting the thing) remains unfulfilled, *séna7* on a *cuz’*-proposition conveys that the expected outcome of the pre-state of an eventuality happening (the eventuality actually happening) remains unfulfilled.

The reader may have noticed that the *=kelh + séna7* data involve present evaluation times (‘will’, not ‘would’-readings), while the *cuz’ + séna7* data involve past evaluation times (‘was going to’, not ‘is going to’ readings). Our analysis predicts that *=kelh* cases can also, in a rich enough context, allow past evaluation times, with readings such as ‘the event described in *p* was predicted to happen, in spite of *q*.’ This is correct, as shown in (109).20Our analysis also technically predicts the existence of *cuz’ + séna7* with present evaluation times, but these would be pragmatically odd. They would simultaneously assert that an event is going to happen, and convey that something unexpected will prevent that event from happening.

(109)

**Table:** 

Context: Julie’s baby boy was frail when he was just born. Nevertheless …				
*Zewát-en-as*	*kwas*	*gélgel=**kelh***	***séna7***	*ku=píxem’*
know-dir-3erg	det+nmlz+ipfv+3poss	strong=**fut**	**cntr**	det-hunt
*(lh=ri<7>p=ás).*					
(comp=grow<inc>=3sbjv)					
‘She knew he would be a powerful hunter (when he grew up).’				
*p: He would be a powerful hunter*		*q: He was weak*		
Consultant’s comment: “Woman’s intuition.”				

We have shown in this section that *séna7* gives rise to different interpretations with the two markers of futurity, *=kelh* versus *cuz’.* With *=kelh,* the truth conditions are that the prejacent event will happen, and *séna7* conveys that something else will happen which is not expected to simultaneously be true (‘*p* will/would happen, in spite of *q*’). With *cuz’,* the truth conditions are that the prejacent event was planned to happen, and *séna7* conveys that counter to expectations, it didn’t happen after all (‘*p* was going to happen, but *q* happened instead’). These are exactly the readings predicted by Glougie’s (2008) analysis of *=kelh* and *cuz’* as a future-oriented modal and a prospective aspect, respectively. This provides a very clean diagnostic for the distinction between futures (which place the evaluation time before the reference time), and prospective aspects (which place the reference time before the event time).21
Copley and Harley (2014) make very similar observations about the interaction of the Tohono O’odham frustrative *cem* with prospective aspect (although they use a different analysis involving the notion of forces, and they do not compare prospective aspect with futures).


The ability of *séna7* to diagnose the semantics of prospective aspect leads to a further result: it enables us to identify a subset of motion verbs in St’át’imcets which must be analyzed as containing prospective semantics. We discuss this in the next sub-section.

### 4.2 *Séna7* as a diagnostic for prospective aspect: extension to motion verbs

St’át’imcets has five motion verbs which can be used as auxiliaries as well as main predicates, and which form the paradigm in Table 2 (adapted from Davis 2017; see also van Eijk 2013).

**Table 2:** Motion verbs.

	telic	atelic
motion towards speaker	*t’iq* ‘get here’	*ts7as* ‘come (here)’
motion away from speaker	*tsicw* ‘get there’	*nas* ‘go (there)’
motion ‘along’	*t’ak* ‘go along’	

Examples of each motion verb are given in (110)–(115), from Davis (2017, Ch. 16). (There are two examples for *t’ak* ‘go along’, as it does not specify the direction towards or away from the speaker.) As discussed by Davis, the different tenses used to translate *t’iq* ‘get.here’ and *tsicw* ‘get there’ (past) versus *ts7as* ‘come’ and *nas* ‘go’ (present) do not reflect a real tense effect. They are the default interpretations when combining telic versus atelic predicates with the null non-future tense (Matthewson 2006b).

(110)

**Table:** 

***T’íq**=wit*	*e=ts7á*	*sát’=a*	*lhl=áku7*	*lh7ús=a.*
**get.here**=3pl	to=deic	Lillooet=exis	from=deic	Lh7us=exis
‘They came here to Sat’ from over there at Lh7us.’

(111)

**Table:** 

***Tsícw**=wit*	*áku7*	*lh7ús=a*	*lhel=ts7á*	*sát’=a.*
**get.there**=3pl	deic	Lh7us=exis	from=deic	Lillooet=exis
‘They went over there to Lh7us from here at Sat’.’

(112)

**Table:** 

***Ts7ás**=wit*	*e=ts7á*	*sát’=a*	*lhl=áku7*	*lh7ús=a.*
**come**=3pl	to=deic	Lillooet=exis	from=deic	Lh7us=exis
‘They are coming here to Sat’ from over there at Lh7us.’

(113)

**Table:** 

***Nás**=wit*	*áku7*	*lh7ús=a*	*lhel=ts7á*	*sát’=a.*
**go**=3pl	deic	Lh7us=exis	from=deic	Lillooet=exis
‘They are going over there to Lh7us from here at Sat’.”

(114)

**Table:** 

***T’ák**=wit*	*e=ts7á*	*sát’=a*	*lhl=áku7*	*lh7ús=a.*
**go.along**=3pl	to=deic	Lillooet=exis	from=deic	Seton=exis
‘They came to Sat’ from Lh7us.’^22^				

22 Some consulatants prefer *t’ak* to refer to motion away from the speaker; for these speakers, examples like (114) are degraded compared to examples such as (115). This extra complication has no effect on telicity, however: see Footnote 24.

(115)

**Table:** 

***T’ák**=wit*	*áku7*	*lh7ús=a*	*lhel=ts7á*	*sát’=a.*
**go.along**=3pl	deic	Seton=exis	from=deic	Lillooet=exis
‘They went to Lh7us from Sat’.’				

When we add *séna7* to sentences containing telic motion verbs, nothing unexpected happens. Like the other achievement predicates discussed in Section 2.2, *t’iq* ‘arrive’ and *tsicw* ‘get there’ retain their culmination. *Séna7* indicates some unexpected happening, such as the failure of the result state to hold or the failure to meet the person one was intending to visit.

(116)

**Table:** 

***T’íq**=k’a*	***séna7,***	*t’u7*	*cw7aoz*	*kwas*	*wá7*	*lhkúnsa.*
**get.here**=epis	**cntr**	but	neg	det+nmlz+ipfv+3poss	be	now
‘He must have arrived, but he’s not there now.’

(117)

**Table:** 

***T’íq**=ti7*	***séna7**,*	*t’u7*	*cw7aoz*	*kwa*	*wá7.*
**get.here**=dem	**cntr**	but	neg	det+ipfv	be
‘He arrived but there was nobody home.’

(118)

**Table:** 

***Tsícw**=kan=t’u7*	***séna7**,*	*t’u7*	*cw7it*	*i=n-száyten=a.*
**get.there**=1sg.sbj=excl	**cntr**	but	many	pl.det=1sg.poss-business=exis
‘I went, but I had too many things to do.’				
Consultant’s comment: “He went, but didn’t stay.”

(119)

**Table:** 

***Tsicw**=kan=tu7*	***séna7**,*	*t’u7*	*kan*	*páqu7-min*	*kwenswá*
get.there=1sg.sbj=dist	**cntr**	but	1sg.sbj	afraid-rlt	det+1sg.poss+nmlz+ipfv
*s-lheqw.*						
stat-ride						
‘I went, but I’m scared to ride horses.’					
*p:*	*I got there*	*q:*	*I didn’t ride*			

The non-cancelability of the culmination with *t’iq/tsicw* and *séna7* is illustrated in (120)–(121).

(120)

**Table:** 

# ***T’íq**=t’u7*	***séna7,***	*t’u7*	*qacw•cw-áwlh*	*nilh*	*s=p’an’t=s*
**get.here**=excl	**cntr**	but	break•fre-vehicle	cop	nmlz=return=3poss
* úxwal’.*
go.home
‘She arrived, but her car broke down so she went home.’						
Consultant’s comment: “Change *t’iq* to *ts7as* [‘come’]; then okay.”

(121)

**Table:** 

# ***Tsícw**=ti7*	***séna7***	*áta7*	*lil’wat7úl=a,*	*t’u7*	*cw7áoy=t’u7*
**get.there**=dem	**cntr**	deic	Lil’wat7úl=exis	but	neg=excl
* kw=s=tsícw•ecw=s.*							
det=nmlz=get.there•fre=3poss								
‘She got to Lil’wat7úl, but she didn’t get there.’					
Consultant’s comment: “These two [*tsicw* and *séna7*] are against each other.”					

*Ts7as* ‘come’ and *nas* ‘go’ show a different pattern. As they are atelic, they allow an interpretation where the subject fails to reach her destination, as in (122)–(124) (which contrast minimally with (120)–(121)).23The progressive/imperfective in the English translations of these examples is not present in the original; these St’át’imcets motion verbs are crucially atelic and allow the destination not to be reached, even in the perfective aspect. However, they also allow an interpretation which is not available for ordinary activity predicates: that no motion took place. This is illustrated in (125)–(126), and it suggests that *ts7as* and *nas* contain prospective semantics. Notice that (118) and (126) form a minimal pair with different interpretations.

(122)

**Table:** 

***Ts7ás**=t’u7*	***séna7,***	*t’u7*	*qacw•cw-áwlh*	*nilh*	*s=p’an’t=s*
**come**=excl	**cntr**	but	break•fre-vehicle	cop	nmlz=return=3poss
*úxwal’.*
go.home
‘She was coming, but she broke down and went back home.’

(123)

**Table:** 

***Ts7ás**=ti7*	***séna7***	*éts7a*	*sát’=a,*	*t’u7*	*cw7áoy=t’u7*
**come**=dem	**cntr**	deic	Lillooet=exis	but	neg=excl
*kw=s=t’iq=s.*		
det=nmlz=get.here=3poss		
‘She was coming to Lillooet, but she never made it.’

(124)

**Table:** 

***Nás**=ti7*	***séna7***	*áta7*	*lil’wat7úl=a,*	*t’u7*	*cw7áoy=t’u7*
**go**=dem	**cntr**	deic	lil’wat7úl=exis	but	neg=excl
*kw=s=tsícw•ecw=s.*			
det=nmlz=get.there•fre=3poss			
‘She was going to Lil’wat7úl, but she didn’t get there.’

(125)

**Table:** 

***Ts7ás**=kan*	***séna7**,*	*t’u7*	*cw7aoz-wíl’c.*
**come**=1sg.sbj	**cntr**	but	neg-become
‘I was coming, but I decided not to.’			
(Alexander et al. in prep.)

(126)

**Table:** 

***Nás**=kan=t’u7*	***séna7**,*	*t’u7*	*cw7it*	*i=n-száyten=a.*
**go**=1sg.sbj=excl	**cntr**	but	many	pl.det=1sg.poss-business=exis
‘I was going, but I had lots of things to do, so I didn’t go.’				

As expected, consultants freely accept minimal pairs involving different contextually provided contrasting propositions, either involving noncompletion of the motion event (127a), failure of the result state to hold (128a), or a complete failure to move ((127b), (128b)).

(127)

**Table:** 

***Nás**=ti7*	***séna7***	*áta7*	*lil’wat7úl=a,*	*t’u7*	*cw7áoy=t’u7*
**go**=dem	**cntr**	deic	Lil’wat7úl=exis	but	neg=excl
*kw=s=tsícw•ecw=s …*			
det=nmlz=get.there•fre=3poss			
‘She went to Lil’wat7úl, but she didn’t get there … ’

a.

**Table:** 

*qacw•cw-áwlh=tu7*	*láta7*	*stéq=a.*
break-•fre-vehicle=dist	there	Duffy.Lake=exis
‘her car broke down at Duffy Lake.’

b.

**Table:** 

*aoz*	*kw=s=ka-qwéts-s-a*	*ta=káoh=a.*
neg	det=nmlz=circ-move-caus-circ	det=car=exis
‘she couldn’t get the car started.’

(128)

**Table:** 

Context: You’re expecting someone.					
***Ts7ás**=ti7*	***séna7,***	*t’u7*	*cw7aoz*	*kwas*	*wá7: …*
**come**=dem	**cntr**	but	neg	det+nmlz+ipfv+3poss	be
‘She was coming, but she isn’t here: …’

a.

**Table:** 

*qwatsats=k’a=wí7=tu7*	*múta7.*
leave=epis=emph=dist	again
‘she must have left again.’

b.

**Table:** 

*wá7=k’a*	*s-t’al*	*l=ta=tsítcw-s=a.*
be=epis	stat-stop	prep=det=house-3poss=exis
‘she must have stayed home.’

The behavior of *ts7as* ‘come’ and *nas* ‘go’ matches that of the prospective aspect *cuz’* as discussed above: unlike other predicates, they allow an interpretation with *séna7* where the prejacent event does not take place. We conclude that they have a reading as prospective aspects.

The fifth motion verb, *t’ak* ‘go along’, is partially similar to *ts7as* ‘come’ and *nas* ‘go’, and partially similar to *t’iq* ‘arrive’ and *tsicw* ‘get there’: it is atelic, but non-prospective. This shows that the two features – (a)telicity and (non-)prospectivity – are separable. Example (129) shows that *t’ak* is atelic (the motion does not have to reach a final destination), and (130) shows that *t’ak* is non-prospective (the motion cannot fail to start at all).

(129)

**Table:** 

***T’ák**.=wit*	***séna7***	*e-ts7á*	*sát’=a*	*lhl=áku7*	*lh7ús=a,*	*t’u7*
**go.along**=3pl	**cntr**	to=deic	Lillooet=exis	from=deic	Seton=exis	but
*cw7áoy=t’u7*		*kw=s=t’íq=i.* ^24^							
neg=excl		det=nmlz=get.here=3pl.poss							
‘They were coming to Sat’ from Lh7us, but they never got here.’						

24 The consultant judges this example as slightly degraded, but his comments suggest that the issue is not the atelicity of *t’ak,* but the fact that *t’ak* prefers motion ‘along’ or ‘by’, and if the motion is towards the speaker as in (129), the preferred motion verb would be *ts7as* ‘come’; see Footnote 22. The consultant’s full comments on (129) are: “I think I’ll let that go. They were going to Lillooet, but they never made it. Better with *ts7as*. Actually, to me, *t’ak* is if they’re going by, *náswit* [*nas* + 3pl] if they’re going, *ts7as* if they’re coming.”

(130)

**Table:** 

# ***T’ák**=kan*	***séna7**,*	*t’u7*	*cw7aoz-wíl’c,*	*cw7áoy=t’u7*
**go.along**=1sg.sbj	**cntr**	but	neg-become	neg=excl
*kw=n=s=qwatsáts.*			
det=1sg.poss=nmlz=leave			
‘I went along, but I didn’t, I didn’t leave.’					
Consultant’s comment: “Had to have set out.” Corrected to … *cw7aoys t’u7 kw nstsícwecw* ‘I didn’t get there.’					

A minimal triplet contrasting the three motion verbs which allow motion away from the speaker is given in (131). Telic, non-prospective *tsicw* ‘get there’ entails that the motion was completed; atelic, prospective *nas* ‘go’ allows no motion at all, and atelic, non-prospective *t’ak* ‘go along’ entails that some motion took place but does not require that the destination is reached.

(131)

**Table:** 

Context: You were meant to be going to a gathering.

a.

**Table:** 

***Tsícw**=kan=t’u7*	***séna7,***	*t’u7*	*cw7it*	*i=n-száyten=a.*
get.there=1sg.sbj=excl	**cntr**	but	many	pl.det=1sg.poss=doings=exis
‘I got there, but I had a lot to do.’				
Consultant’s comment: “It says you went, because of *tsícwkan.”*

b.

**Table:** 

***Nás**=kan=t’u7*	***séna7**,*	*t’u7*	*cw7it*	*i=n-száyten=a.*
go=1sg.sbj=excl	**cntr**	but	many	pl.det=1sg.poss=doings=exis
‘I was going to go, but I had a lot to do.’				
Consultant’s comment: “Didn’t go.”

c.

**Table:** 

***T’ák**=kan=t’u7*	***séna7**,*	*t’u7*	*cw7it*	*i=n-száyten=a.*
**go.along**=1sg.sbj=excl	**cntr**	but	many	pl.det=1sg.poss=doings=exis
‘I went, but I had a lot to do.’				
Consultant’s comment: “He was going, but he came back.”

In summary, not only are the predictions of our analysis of *séna7* confirmed, *séna7* interacts with future time-reference in a predictable way and provides a clear diagnostic for the presence of prospective semantics in a subset of the motion verbs of the language.

## 5 Comparison with other frustratives

In this section we compare *séna7* to similar elements crosslinguistically, and explain why previous analyses are not applicable to *séna7.* We also propose a potential re-analysis of another frustrative marker, Kimaragang *dara*, to make it parallel to our analysis of St’át’imcets *séna7.* 25A related element that we do not discuss is the Hua ‘inconsequential’ clause-type (Haiman 1988). Inconsequential clauses seem to share some uses with frustratives, including the idea of an ‘*as yet* fruitless or vain activity’ (Haiman 1988: 57; emphasis original), and denial of causal succession between two clauses. However, they also have other, unrelated, functions such as signaling a change of speaker in dialogue. Thanks to a reviewer for pointing us to this work.


### 5.1 Tohono O’odham *cem*

A well-known frustrative marker is Tohono O’odham *cem* (Copley 2005; Copley and Harley 2014; Hale 1969; see also Devens 1979 on the cognate in closely related Pima/Akimel O’odham). Copley and Harley (2014: 123) remark that ‘Descriptively speaking, sentences with frustratives can express the fact that the subject intended to do something that is not realized; that [the] subject does something in vain; that a situation is unsatisfactory or does not develop as expected, or that a state does not continue.’ Examples are given in (132)–(133).

(132)

**Table:** 

*Huan*	*’o*	***cem***	*kukpi’ok*	*g*	*pualt.*
Juan	aux-ipfv	**frus**	open	det	door
‘Juan pulled on the door but failed to open it.’					
(Copley and Harley 2014)

(133)

**Table:** 

***Cem***	*’añ*	*ñ-na:tokc.*
**frus**	1sg	1sg-ready
Non-continuation:		‘I was ready but now I’m no longer ready.’				
Unachieved goal:		‘I was ready but you weren’t there.’			
(Copley 2005)				

Copley (2005) argues that *cem(p)* sentences (a) assert that all inertia worlds for the topic situation *s* are worlds in which *p(s),* and (b) presuppose that the actual world is not an inertia world for *s.* Copley and Harley (2014) replace the inertia worlds analysis with an approach involving *forces* (see also Copley and Harley 2015). Forces are inputs of energy which act on situations. An *efficacious* situation is one whose normal expected result (given the forces in the situation) obtains (see Copley and Harley’s paper for the full formal definition).26See Louie (2014) for an analysis of efficacy in terms of modality, without the need for forces. Louie applies efficacy in the analysis of actuality entailments in Blackfoot. They argue that *cem(p)* sentences presuppose that the topic situation *s* is not efficacious (i.e., its normal result does not obtain). Their denotation for *cem* is given in (134).

(134)

**Table:** 

[[ *cem* ]] = λs λp . p(s)	
presupposed: s is not efficacious
(Copley and Harley 2014: 139)

According to this denotation, *cem(p)* is truth-conditionally identical to *p* (just as we have proposed for *séna7*)*.* This feature of Copley and Harley’s analysis might initially seem to clash with the characterization given above that *cem* is licensed by contexts in which the subject intended to do something that is not realized, or with example (132) in which Juan did not manage to open the door. However, the role of aspectual morphology is crucial: when the clause is perfective, *cem*’s prejacent *is* actualized, so sentences like (132) which allow non-realization are necessarily in the imperfective. A more literal translation of (132) would presumably be ‘Juan was opening the door,’ which is truth-conditionally compatible with him not opening it.

Apart from the technical tools used (forces and efficaciousness as opposed to quantification over possible worlds), the other difference between Copley and Harley’s analysis of *cem* and ours of *séna7* is that the former relies on the normal progression of situations and their expected results or outcomes.27There may be a further difference relating to the enforcement of past temporal reference with *cem*, but it is not clear whether this is contributed by *cem*’s semantics or is a pragmatic effect, so we set this aside; see Hale (1969), Devens (1979), Copley (2005), Copley and Harley (2014). We have argued on the basis of examples like (90)–(98) above (e.g., ‘The potatoes got cooked but the chicken didn’t’) that *séna7* conveys unexpectedness but does not always rely on causes and effects. As Copley and Harley do not discuss data like (90)–(98), it is difficult to be sure whether (failed) causation is a crucial requirement of *cem.*

A related issue is the effect of *cem* on imperfective accomplishments. Copley and Harley write that ‘In the case of the imperfective, a sentence with *cem* as in [(132)] conveys that Juan does something to open the door, but the door does not open’ (2014: 148). This is in line with the idea that *cem* signals the failure of the normal, expected outcome of pulling on a door: that it opens. However, *séna7* in a parallel case can not only have the non-culmination interpretation, but can also convey a non-causally-related unexpected event. This is shown in (135).

(135)

A:

**Table:** 

*Kánem*	*s=cw7aoy=s*	*kw=s=tsicw=s*
why	nmlz=neg=3poss	det=nmlz=get.there=3poss
*ats’x-en-túmulh-as*	*kw=s-Sally*	*i=zánucwem=as?*		
see-dir-1pl.obj-3erg	det=nmlz-Sally	when.pst=year=3sbjv		
‘Why didn’t Sally come to visit us last year?’

B:

**Table:** 

***Wá7**=tu7*	***séna7***	*mets-en-ás*	*ta=púkw=a,*	*t’u7*
**ipfv** =dist	**cntr**	write-dir-3erg	det=book=exis	but
*ús-ts-as*		*i=plán=as*		*tsem’p.*				
throw.out-caus-3erg		when.pst=already=3sbjv		finished				
‘She was writing a book, but she threw it away when it was finished.’

Copley and Harley do not provide data like (135) involving imperfective accomplishments which eventually culminate, but involve other unexpected eventualities*.*

Beyond the analysis of *cem*, Copley and Harley have the larger goal of partially unifying frustratives with non-culminating accomplishments; the latter were discussed in Section 2.2 and are illustrated again in (136) for St’át’imcets.

(136)

**Table:** 

*K’ul’-ún’=lhkan*	*ti=ts’lá7=a,*	*t’u7*	*áoy=t’u7*	*kw=tsukw=s.*
make-dir=1sg.sbj	det=basket=exis	but	neg=just	det=[nmlz=]finish=3poss
‘I made the basket, but it didn’t get finished.’				
(Bar-el et al. 2005: 90)				

Copley and Harley claim that frustratives and non-culminating accomplishments both involve non-efficacy, but differ in whether this is enforced or only allowed. Frustratives presuppose that the topic situation is non-efficacious (as in (134)). Culminating accomplishments presuppose that the topic situation *is* efficacious; it is therefore entailed that the end result of the net force (the culmination) actually occurs. Non-culminating accomplishments fail to presuppose this, and therefore allow the absence of culmination.

Using data from the Austronesian language Kimaragang, Kroeger (2017) argues against Copley and Harley’s efficacy-based partial unification of frustratives and non-culminating accomplishments. One empirical argument advanced by Kroeger is that in Kimaragang, the frustrative marker (which according to Copley and Harley would presuppose *non-efficacy*) can co-occur with non-volitive marking, which enforces culmination on accomplishments and which therefore according to Copley and Harley would presuppose *efficacy*.

The same is true in St’át’imcets. There is a small class of transitive predicates in St’át’imcets which test as achievements, due to the fact that their instantaneous or near-instantaneous running time prevents them from being initiated without also culminating (see Footnote 12). As shown in (137)–(138), members of this class can co-occur with *séna7* (see also (66)–(67) in Section 2.2.2)*.* This would result in a fatal clash of presuppositions in Copley and Harley’s account.28A reviewer suggests that these facts could be dealt with in Copley and Harley’s model, by saying that the efficacy requirement of the Kimaragang non-volitive or the St’át’imcets causative applies to a smaller situation than the non-efficacy requirement of the frustrative. This would allow culmination to be enforced, but some other unexpected outcome to happen in a larger situation.We agree with this, and therefore our argument here is not a knock-down one. However, this idea would require some revisions to Copley and Harley’s analysis (which as it stands, applies both the (non-)efficacy requirements to the topic situation), and it would somewhat weaken the strong parallel they draw between non-culminating accomplishments and frustratives: ‘Non-culminating accomplishments do not require any special construction or morphology to indicate the failure of a normal or expected event … In other languages, a separate construction is dedicated to such failures: the frustrative’ (2014: 134).

(137)

**Table:** 

*Kwís-**ts**=kan*	***séna7***	*ta=xmánk=a*	*xétsem*	*l=ta=n-sq’wáxt=a,*
fall-**caus** =1sg.sbj	**cntr**	det=heavy=exis	box	prep=det=1sg.poss-foot=exis
*t’u7*	*áoy=t’u7*	*kw=n=s=ka-qácw-cen-a.*				
but	neg=excl	det=1sg.poss=nmlz=circ-break-foot-circ				
‘I dropped a heavy box on my foot, but my foot didn’t break.’

(138)

**Table:** 

*Pelp’-**s**=kán*	***séna7***	*ta=nqláw’ten=a,*	*t’u7*	*pún=lhkan*	*múta7.*
lost-**caus** =1sg.sbj	**cntr**	det=wallet=exis	but	find+dir=1sg.sbj	again
‘I lost my wallet but then I found it again.’					

A second argument against unifying frustratives with non-culminating accomplishments, again originally due to Kroeger (2017), is that the available interpretations for the two phenomena are quite different. In St’át’imcets, accomplishments without *séna7 only* allow an event to be ‘non-normal’ in the sense that its culmination need not take place. *Séna7,* in contrast, allows a broader range of interpretations, as we showed in Section 2: not only failure to culminate, but also other unexpected outcomes, failure of the result state to hold, or that the event did not happen well.

It is not easy to demonstrate via negative evidence that accomplishments without *séna7* only license unexpected interpretations which involve non-culmination, because any simple predication *not* containing a frustrative can be followed by a clause saying that the event had an unexpected outcome or was not successful. However, indirect evidence comes from consultants’ responses to monoclausal, out-of-the-blue sentences containing only accomplishment verbs, as opposed to their responses to *séna7*-sentences. We illustrate this in (139)–(140).

In (139), an accomplishment predicate, either with or without *séna7,* can be followed by a query about whether the culmination was reached. No special context is needed in order for the possibility of non-culmination to seem natural. The presence of *séna7* merely makes this more probable, as indicated by the consultant’s comment on the *séna7* version.

(139)

**Table:** 

Context: You hired your nephew to work on things around your land. He comes to you at the end of the day.			
Nephew:	*Máys-**en**=lhkan*	*(**séna7**)*	*ta=q’láxan=a.*
	get.fixed-**dir** =1sg.sbj	(**cntr**)	det=fence=exis
	‘I fixed the fence.’		
You:	*Tsúkw-s=kacw=ha?*		
	finish-caus=2sg.sjb=q		
	‘Did you finish?’		
Consultant’s comment on version with *séna7:* “That *séna7* in there makes you wonder what seems to be wrong, so you ask him if he’s really finished.”			

In (140), on the other hand, the responder asks more generally “What seems wrong?”, rather than specifically asking about non-culmination. As in (139), the consultant comments on the role of *séna7* in conveying that something went wrong, but unlike in (139), the consultant remarks that the non-*séna7* version needs a visual cue that something went wrong. This difference between non-culmination (as in (139)) and general unexpectedness or failure (as in (140)) supports the idea that the two phenomena of non-culminating accomplishments and frustrativity should not be unified in terms of a single notion of ‘nonefficacy’.

(140)

**Table:** 

Context: As in (139).			
Nephew:	*Máys-**en**=lhkan*	***(séna7)***	*ta=q’láxan=a.*
	get.fixed-**dir** =1sg.sbj	(**cntr**)	det=fence=exis
	‘I fixed the fence.’		
You:	*Stam’*	*kwa*	*eswátem?*
	what	det+ipfv	wrong
	‘What seems wrong?’				
Consultant’s comment on version without *séna7:* “Yep, if you’re looking at him and you see something wrong.”			
Consultant’s comment on version with *séna7:* “That *séna7* makes it sound like there’s something wrong.”			

A final argument against the unification of *séna7* and non-culminating accomplishments, not discussed by Kroeger (2017), relies on morphological evidence. Recall that according to Copley and Harley, non-culminating accomplishments presuppose nothing about efficacy, and it is culminating accomplishments which bear a presupposition (that the topic situation is efficacious). Thus, ‘[t]he absence of a culmination is the basic case’ (2014: 135). In support of this, Copley and Harley claim that ‘non-culminating accomplishments do not require any special construction or morphology to indicate the failure of a normal or expected outcome to occur,’ and that this ‘allows us to treat cases of defeasible causation straightforwardly, instead of first generating and subsequently undoing a causative entailment’ (2014: 134).

This analysis makes the wrong predictions for Salish languages. In St’át’imcets and other languages in the family, non-culminating accomplishments *do* need special morphology (directive transitivizers). The bare root is always a telic achievement (as shown in Section 2.2), so the morphological evidence suggests that we *do* need to first generate a culmination and then undo it. This in turn suggests that we need to assign semantic content both to *séna7* and to the directive transitivizer, and since these two elements are morphologically distinct, there is no morphological argument that they should be partially unified semantically.

### 5.2 Kimaragang *dara*

In this section we show that the Kimaragang frustrative particle *dara,* discussed by Kroeger (2017), is very similar to St’át’imcets *séna7.* We further argue that the two may be even more similar than Kroeger’s own analysis of *dara* suggests, and propose a potential re-analysis along the lines we have proposed for *séna7.*

*Dara* is found in a range of contexts, including cases of unfulfilled desires or intentions, failed attempts, former states that no longer obtain, states that do not lead to expected results, things done in vain, and counterfactual conditionals (Kroeger 2017: 2). Two of these uses are shown in (141)–(142).

(141)

**Table:** 

*N-o-sii-Ø*	*ku*	*no*	***dara***	*it=tasu*	*nga’*	*n-iit-an*	*oku=i’*
pst-nvol-shoo-ov	1sg	already	**frust**	nom=dog	but	pst-bite-dv	1sg=emph
‘I said *Shii!* to the dog, but I got bitten anyway.’							
(Kroeger 2017: 3)

(142)

**Table:** 

*Waro*	***dara***	*siin*	*ku*	*nga’*	*n-i-baray*	*ku*	*dot=tutang.*
exist	**frust**	money	1sg.gen	but	pst-iv-pay	1sg.gen	acc=debt
‘I did have money but I used it to pay off my debt.’						
(Kroeger 2017: 3)							

Kroeger unifies all the uses of *dara* as expressing ‘frustrated expectation or intention’ (2017: 1). He proposes that *dara* asserts that some salient proposition is true in all optimal (i.e., highest-ranked) accessible worlds, and presupposes that the actual world is nonoptimal in the relevant respects (thus, that the salient proposition is false). The unrealized proposition can be *dara*’s prejacent, or if this is not possible, then it ‘may be inferred from context, and typically describes a successor event or result state of the situation described in the *dara* clause’ (Kroeger 2017: 15). For example, (141) asserts that in all the most optimal worlds, the speaker isn’t bitten, and presupposes that the actual world is non-optimal, so the speaker did get bitten.29This is very similar to Copley’s (2005) earlier analysis of Tohono O’odham *cem,* as Kroeger himself points out (2017: 15).


In Kroeger’s analysis, *dara-*clauses make either epistemic or bouletic modal claims. (141) and (142) are epistemic: they have ‘frustrated expectation’ readings. They assert that in all stereotypical worlds compatible with the speaker’s knowledge, the dog leaves the speaker alone/the speaker still has money, and at the same time they presuppose that these optimal propositions are false: the dog did not leave the speaker alone, and the speaker no longer has money.

A bouletic (‘frustrated intention’) case is shown in (143). Here, the optimal but false salient proposition is the prejacent itself. According to Kroeger’s analysis, the sentence asserts that in all worlds that are compatible with the relevant circumstances and in which Mother’s desires or intentions are fulfilled, she binds the fish trap; it also presupposes that she does not bind it.

(143)

**Table:** 

*Momolit*	*i=iyay*	*di=bubu*	***dara***	*nga’*	*asot*	*wakaw.*
av.tr1-bind	nom=mother	acc=fish.trap	**frust**	but	not.exist	rattan
‘Mother would/wants to bind the fish trap (that she built), but she is out of rattan.						
(Kroeger 2017: 16)						

Kroeger’s analysis and ours are similar in that they both invoke modality and use no extra theoretical tools beyond the standard ones of restricted quantification over possible worlds. However, there are two important differences between the analyses.

First, Kroeger’s analysis employs modality in the *at-issue* truth conditions: a *dara-*clause makes a modal assertion. This allows *dara*’s prejacent to be false in the actual world. In contrast, we have argued that *séna7*’s contribution is not-at-issue and has no effect on truth conditions; thus, *séna7*’s prejacent is entailed to be true in the actual world. Second, Kroeger allows intention readings (with a circumstantial modal base and a bouletic ordering source), while our analysis is purely epistemic: the only factor for *séna7* is speaker expectation.

Nothing theoretically would rule out frustratives varying in these ways cross-linguistically. It is already known that languages encode a range of different fine-grained modal distinctions. Moreover, what is conveyed in the at-issue realm by one element can be conveyed in the not-at-issue realm by another element in the same or another language. For example, DeVeaugh-Geiss (2014) and Zimmermann (2018) argue that the German particles *wohl* and *schon* contribute not-at-issue modal semantics corresponding respectively to the at-issue modal elements *werden* and *eher* in the same language*.*

However, we suspect that *dara* may in fact be fully compatible with our analysis of *séna7,* which would be an interesting result, as St’át’imcets and Kimaragang are unrelated languages. We propose that a unified analysis can be given for *séna7* and *dara* while still capturing the apparent empirical differences between the two frustratives.

With respect to whether two different modal flavors (epistemic vs. bouletic) are required, we observe that in both languages, the facts are the same, namely that both expectation-related and intention-related interpretations are available. However, we propose that a unified epistemic analysis can capture the facts. By adopting one extra assumption – that the expected outcome of an intention is that the intention is fulfilled – we can reduce the failure of intention cases to failure of expectation cases. In fact, we already made this assumption in Section 2.1, following Copley and Harley’s (2014) Law of Rational Action, which states that a volitional agent with a desire will act as a force which ceteris paribus will result in the desired situation coming about.30
Overall (2017) similarly proposes that the core meaning of frustratives is always epistemic. His definition of frustratives differs from our analysis, however, in also including the notion of an unrealized outcome. We have argued that *séna7* does not always rely on the notion of an expected outcome, but instead on an unexpected co-occurrence of any two true propositions.


An apparently more substantial obstacle to the unification of *séna7* and *dara* is that *séna7(p)* entails *p* (as we argued extensively above), while *dara(p)* does not (as for example in (143)). This is what leads Kroeger to adopt an at-issue modal semantics for *dara.* However, there are some significant exceptions to Kroeger’s claim, where *p is* in fact entailed by *dara(p).* These include cases where the prejacent clause is marked for past tense, as well as predicates which describe states in the past or present. In these cases, *dara* entails its prejacent*,* just like *séna7* does*.* An example is given in (144): we see that the past tense-marked version of the sentence does not allow a non-realized interpretation with *dara.*

(144)

a.

**Table:** 

		*Patay-on*	*ku*	***dara***	*ilo’*	*masalong*	*nga’,*	*tiniag*	*oku*	*di=ama.*
		kill-ov	1sg	**frust**	that	cobra	but	pst.forbid.ov	1sg	gen=father
		‘I was going to kill that cobra, but Father forbade me.’

b.

**Table:** 

?*	*P<**in**>atay-Ø*	*ku*	***dara***	*ilo’*	*masalong*	*nga’*	*tiniag*	*oku*
	< **pst** >kill-ov	1sg	**frust**	that	cobra	but	pst.forbid.ov	1sg
	*di ama.*
	gen=father
	(Kroeger 2017: 17)

Kroeger’s account of this ‘realis’ effect with *dara* on past-inflected eventives is that ‘we see a kind of shift in the function of the tense morphology: it marks a contrast between past versus non-past time reference in main clauses and similar contexts, but realis versus irrealis in *dara* clauses (2017: 18).’ However, simply adding realis marking to a clause containing an at-issue modal does not actually achieve the effect of requiring the prejacent proposition to be true in the actual world (this is true whether the realis semantically scopes over or under the modal). To have the intended effect, the realis contribution of the past tense marker would have to actively cancel *dara*’s at-issue modal contribution, something which would be compositionally problematic. In addition, postulating a semantic ambiguity in the contribution of the past/realis marker is less desirable conceptually than having a unified analysis. Finally, the proposed realis reading of the past marker does not account for the realis effect with non-future statives. For these, Kroeger writes that he ‘do[es] not have a good explanation’ (2017: 20).

Our proposed alternative analysis of *dara,* which leads to further predictions about Kimaragang which await testing, is that it has an identical semantics to *séna7.* Under this analysis, the empirical difference between the languages – the fact that *dara* appears to allow false prejacents and *séna7* does not – derives not from a difference between the two frustrative markers, but from independent differences in the tense/aspect systems of the languages.

The temporal systems of the two languages are in fact different: St’át’imcets has a future/non-future tense system (with non-future being phonologically null; Matthewson 2006b), while Kimaragang has a past/non-past tense system for eventives (with non-past being phonologically null), while statives are not normally marked for tense (Kroeger 2017). Thus, temporally unmarked predicates in St’át’imcets can only be interpreted as having past or present time reference, while temporally unmarked eventive predicates in Kimaragang allow present or future time reference. If Kimaragang unmarked eventive predicates allow future time reference, then *dara-*clauses with these unmarked predicates could in effect be parallel to St’át’imcets *séna7*-clauses with prospective aspect *cuz’.* The apparent ‘unrealized’ status of *dara*’s prejacent would then derive not from *dara* itself, as in Kroeger’s analysis, but from the inherent futurity/unreality of the prejacent, as in our analysis of St’át’imcets *séna7-*clauses with prospective aspect.

The behavior of stative predicates in Kimaragang could potentially also fall out from this reanalysis, since stative predicates interpreted in the present or past require realis interpretations with *dara* (Kroeger 2017: 20)*.* This follows if (a) *dara(p)* entails *p,* as in our reanalysis, and (b) statives, unlike eventives, do not allow prospective or future interpretations without overt temporal marking. Kroeger does not give examples of stative predicates with future interpretations, so further research is required to determine whether this prediction is upheld.

This proposed reanalysis of the Kimaragang facts has an additional advantage: it does away with the presupposition that the optimal proposition is unrealized. This is a welcome result because in many cases (including (141) and (142)), the postulated presupposition is overtly introduced by, or at least implicated by, a follow-up clause.31In (141), Kroeger’s proposed presupposition is that the speaker got bitten; this is overtly stated. The presupposition in (142) – that the speaker no longer has money – is implicated by the second asserted clause. Presuppositions by definition are assumed to already be in the common ground and therefore are not usually overtly stated (cf. *#The King of France is bald, and there is a unique King of France*).32The proposed reanalysis would also bring Kimaragang into parallel with the frustratives discussed by Overall (2017), for which he argues that ‘The state of affairs (proposition *p*) expressed by the marked predicate is asserted’ (2017: 479–480).


Before leaving Kimaragang, we need to reiterate that we are fully in agreement with what we take to be the main point of Kroeger’s paper: that the meaning of frustratives like *dara* is not unifiable with the meaning of non-culminating accomplishments, pace claims by Copley and Harley (2014); see discussion in the previous sub-section.

### 5.3 Tagalog AIA (ability/involuntary action)

The final related phenomenon we discuss is ability/involuntary action morphology on Tagalog verbs (Alonso-Ovalle and Hsieh 2017a, 2017b, 2018). The effect of AIA morphology is illustrated in (145)–(146). In (145), the verb has neutral morphology; the sentence simply asserts that Lisa opened the door. In (146), extra meaning is conveyed by the AIA marking.

(145)

**Table:** 

*B<**in**>uks-an*	*ni*	*Lisa*	*ang*	*pinto.*
<prf.**ntl** >open-lv	gen	Lisa	nom	door
‘Lisa opened the door.’

(146)

**Table:** 

***Na**-buks-an*	*ni*	*Lisa*	*ang*	*pinto.*
pfv.**aia** -open-lv	gen	Lisa	nom	door
‘Lisa managed to open the door.’/‘Lisa accidentally opened the door.’							
(Alonso-Ovalle and Hsieh 2017a, 2017b, 2018)					

Alonso-Ovalle and Hsieh (2018) argue that a sentence containing AIA morphology asserts the core proposition *p* (e.g., that Lisa opened the door), and presupposes that, given the facts that *p* is assumed to causally depend on, *¬p* was expected. This analysis shares with our analysis of *séna7* the fact that the core proposition is asserted, and that modality is introduced in the not-at-issue dimension. The modality for AIA morphology relies on a set of worlds defined by a set of causally relevant facts (cf. Kaufmann 2013), plus a stereotypical ordering source.

Although the not-at-issue status of the modality is parallel between Tagalog AIA morphology and St’át’imcets *séna7,* there are also differences which seem to speak against a unified analysis. One major one is that AIA morphology is said to enforce the inherent unexpectedness of the prejacent *p* itself*.* Thus, AIA morphology is inappropriate in (147).

(147)

**Table:** 

# **Naka**-labas	ang	araw.
pfv.**aia**.av-come.out	nom	sun
‘The sun came out.’		
(Alonso-Ovalle and Hsieh 2018: 66)		

We saw in (85) (‘The sun came up, but it was cloudy, so we couldn’t see it’) that St’át’imcets *séna7* is compatible with inherently expected prejacent events. *Séna7* is also possible even in an out-of-the-blue, monoclausal statement about the sun parallel to (147), as long as it is possible to accommodate some salient unexpected second proposition. This is shown in (148).

(148)

**Table:** 

*Ka-cát-q-a*	***séna7***	*ta=snéqwem=a.*	
circ-rise-bottom-circ	**cntr**	det=sun=exis	
‘The sun DID come up.’					
Consultant’s comment: “But maybe cloudy or foggy, so you can’t see it.”					
*p: The sun came up*	*q: You can’t see the sun*	

We have argued that this result follows because *séna7* does not force its prejacent to be inherently expected, but merely marks the unexpected co-occurrence of the prejacent with some other proposition. While the contrast between (147) and (148) is certainly suggestive, it would be interesting to see whether AIA morphology could become acceptable in ‘sun’ cases by making the contrasting proposition salient in the context, for example in a biclausal case like (85).

## 6 Summary, open issues and implications

### 6.1 Summary

The St’át’imcets frustrative *séna7* poses a prima facie analytical challenge, due to the apparently wide range of readings it gives rise to: failure of expected outcome, noncontinuation of an eventuality, unexpected co-occurrence of two eventualities, an eventuality not happening very well, the failure of a result state to hold, the failure of culmination, and even that an event didn’t happen at all (only with prospective aspect).

We have argued that the meaning of *séna7* is best captured by the analysis in (149). According to this, *séna7* takes one semantic and syntactic argument: its prejacent clause. It has no effect on the at-issue truth-conditions of this clause, so an utterance of *séna7(p)* asserts *p.* In the not-at-issue dimension, *séna7* conveys that the discourse context contains a separate salient true proposition *q,* and the speaker does not expect *p* and *q* to both be true. The contrasting proposition *q* can be provided by a subsequent clause, an implicature of asserted material, real world knowledge, or other means; as such, the interpretation of *séna7*-clauses is highly context-dependent.

(149)

**Table:** 

[[ *séna7 (p)* ]]^c,w^ =		
At-issue:	[[ *p* ]]^c,w^
Not-at-issue:	∃q [(q(w) = 1) & ¬∃w’ [w’ ∈ BEST_STEREO(w)_(∩EPIS_sp(c)_(w)): p(w’) = 1 & q(w’) = 1]]	

We have further shown that *séna7* can be used as a diagnostic to tease apart entailments from implicatures, using telicity as a case study: *séna7* helps to distinguish achievements, which have a culmination entailment, from control accomplishments, which only have culmination implicatures. *Séna7* also distinguishes between two ways of expressing future time reference: with the modal clitic *=kelh, séna7* asserts that an event will happen in the future, and conveys that something unexpected will also happen (‘*p will happen, in spite of q*’), whereas with the prospective auxiliary *cuz’,* it is the pre-state of an eventuality which contrasts with a second proposition *q*; the most common interpretation is *‘p was going to happen, but q happened instead’.* This provides a diagnostic for teasing apart futures (which place the reference time after the evaluation time) and prospective aspects (which place the event time after the reference time). Finally, we showed that *séna7* distinguishes motion verbs along both parameters: telic versus atelic (requiring vs. not requiring the reaching of an endpoint) and prospective versus non-prospective (allowing vs. not allowing no motion at all to take place).

Crosslinguistically, we showed that *séna7* yields similar interpretations to other frustratives, including Tohono O’odham *cem,* Kimaragang *dara* and Tagalog AIA morphology. We argued that the differences between *séna7* and *dara* may reduce to independent differences in the temporal systems of the languages; this paves the way for a unified analysis, but requires empirical confirmation in future research. We have in addition argued (following Kroeger 2017, but pace Copley and Harley 2014) that non-culminating accomplishments are fundamentally different from frustratives.

Methodologically, this paper contributes to semantic and pragmatic fieldwork along two dimensions. First, we have shown that rich contextual specification, partially co-created with our language consultants, can yield precise formulations of subtle not-at-issue phenomena such as the meaning of frustratives. Second, we have shown that once their precise contribution is understood, frustratives such as *séna7* can themselves be employed as diagnostic tools to tease apart implicatures and entailments, as we demonstrated in our analysis of non-culminating accomplishments, prospective aspect, and motion verbs in St’át’imcets.

### 6.2 Open issues and implications

An interesting topic for future research is the effect of *séna7* inside questions, as in (150).

(150)

**Table:** 

*Cw7it*	*nelh=s-7ílhen-sw=a.*	*Wá7=lhkacw=ha*	***séna7***	*tayt?*
many	pl.det=nmlz-eat-2sg.poss=exis	ipfv=2sg.sbj=q	**cntr**	hungry
‘You ate lots. Are you really hungry?’				
Consultant’s comments: “If you’re watching somebody eating and they’re eating lots: ‘I wonder where he’s putting it all?’”				

One recent analysis of not-at-issue content inside questions is that of Davis and McCready (2016). They argue that when an expressive element appears in a question, it can operate on whatever is the *true* answer to the question. Applying this idea to *séna7,* we would predict that in (150), the speaker is (a) asking whether the addressee is hungry, and (b) conveying that whatever the true answer is to the question, it is unexpected. This makes sense, since either answer would be unexpected in such a context. If the addressee isn’t hungry, it’s unexpected that they are still eating (as in the consultant’s volunteered context for the utterance). If the addressee *is* hungry, that is unexpected given that they just ate a lot. However, further research is required here.33An interpretation we clearly predict not to exist is one where the contribution of *séna7* scopes under the question operator. That is, we do not expect an interpretation where the speaker is questioning *whether it is unexpected* that the addressee is hungry despite having eaten a lot.


Similarly, future research should extend the analysis of *séna7* to capture its contribution inside imperatives. One example is given in (151).

(151)

**Table:** 

*T’anam’-ílc=malh*	***séna7**!*
try-aut=adhort	**cntr**
‘You better try anyway!’	
Consultant’s comment: “Doesn’t think he can do it.”	

Another interesting area for future systematic investigation is the interaction of *séna7* with the felicity conditions of prior speech acts. The preliminary data we have are compatible with our analysis, under the assumption that the contextually salient proposition *q* can be provided by specific felicity conditions in the discourse context. Examples are given in (152)–(154) for *séna7-*utterances following a command, a question, and an assertion. In each case, *q* is a felicity condition of the preceding speech act.

(152)

A:

**Table:** 

*Úlhcw-slep’=malh!*
enter-firewood=adhort
‘Fetch the firewood!’

B:

**Table:** 

*Qácw•ecw-cen’=lhkan*		***séna7!***	*Sáw-en*	*ku=núkw.*
break•fre-foot=1sg.sbj		**cntr**	ask-dir	det=other
‘But I have a broken leg! Ask somebody else.’			
*p:*	*I have a broken leg*	*q:*	*A believes I can fetch firewood*

(153)

A:

**Table:** 

*S-kenkán*	*kw=s=cin’=s*	*kw=s=we7-án-acw*
stat-how.much	det=nmlz=long.time=3poss	det=nmlz=hold-dir-2sg.erg
*ts7a ku=púkw?*		
this det=book		
‘How long have you had this book?’

B:

**Table:** 

*Snúwa*	***séna7***	*ta=um’-en-ts-ás=a*	*i=klísmes=as!*
2sg.indep	**cntr**	det=give-dir-1sg.obj-3erg=exis	when.pst=Christmas=3sbjv
‘You gave it to me for Christmas!’			
*p:*	*A gave it to me for Christmas*		*q: A doesn’t know how long I’ve had it*

(154)

A:

**Table:** 

*Wa7*	*láti7*	*ta=tsíken=a*	*l=ta=n-lep’-cal-ten-láp=a!*
be	deic	det=chicken=exis	prep=det=loc-dig-act-2pl.poss=exis
‘There is a chicken in your garden!’

B:

**Table:** 

*Lán=t’elh*	***séna7***	*q’em’p*	*máqa7*	*kwas*
already=at.this.time	**cntr**	ten	snow	det+nmlz+ipfv+3poss
*we7-án-em*	*i=tsíken=a.*						
be-dir-1pl.erg	pl.det=chicken=exis						
‘Well, we’ve had chickens for 10 years.’				
*p:*	*We’ve had chickens for 10 years*	*q: A believes I don’t know we have chickens*

There is precedent in the literature for the idea that discourse-sensitive elements like *séna7* can respond to felicity conditions; for example, Egg (2010) and Egg and Zimmermann (2012) propose that German discourse particles can respond not only to propositional content, but to the felicity conditions of speech acts.

Eventually, frustratives like *séna7* should be compared with a broader cross-linguistic set of markers encoding a sense of contrast, including for example conjunctions such as English *but* or *(even) though,* German discourse particles like *doch, zwar* or *schon,* and Russian correction and adversative markers (Jasinskaja 2012; Jasinskaja and Zeevat 2008).34Within the Salish family, there are also other particles which encode contrast. In ʔayʔaǰuθəm (Comox-Sliammon), there is an element *ʔiy* which Reisinger and Huijsmans (2019) analyse (loosely following Hinterwimmer and Ebert 2018 for German *aber* ‘but’) as being defined for a prejacent proposition *φ* only if there is a salient proposition *ψ* which entails *¬φ* in *c*. Two clear differences between *séna7* and *but* or *(even) though* are the fact that *séna7* takes only one syntactic argument, with the other clause provided by the context, and that *séna7* is semantically symmetrical (it can appear on either of the two propositions it relates, with no difference in meaning, as we showed in Section 3.2; this is not the case for *but* or *even though;* see Umbach 2005; Toosarvandani 2014, among many others).

When it comes to German discourse particles which encode contrast like *doch,* an interesting point of comparison with *séna7* is that – at least in many people’s analyses – particles like *doch* presuppose that certain information is in the common ground (for discussion, see Karagjosova 2009; Egg 2010; Grosz 2011, 2020; Zimmermann 2011, among many others). We showed in Section 3.4 that the unexpectedness requirement of *séna7* need only hold for the speaker; it is not a traditional presupposition (although it is in the not-at-issue dimension). This is in line with research showing that St’át’imcets in general lacks presuppositions which refer to the common ground (Matthewson 1998, 2006a, 2009).

A final observation about frustratives crosslinguistically is that they belong to a range of grammatical categories and appear in different syntactic positions. *Séna7* is a sentence-level adverb, *cem* is a pre-verbal particle, *dara* is a second-position clitic and AIA morphology is a paradigm of verbal inflection. This speaks against a possible cartographic approach in which there would be a dedicated position in the syntactic spine where frustrative semantics is located.35Thanks to a reviewer for pointing this out. Instead, it suggests a ‘semantic building blocks’ approach (cf. von Fintel and Matthewson 2008; Hale 1986), whereby small pieces of meaning recur crosslinguistically, sometimes combining with other semantic building blocks inside single morphemes, and they are distributed across different parts of the syntactic architecture.

### 6.3 Conclusion

We have proposed an analysis of St’át’imcets *séna7* which involves only the standard tools used to analyze modal elements, but in the not-at-issue dimension. To capture *séna7*’s contribution, it is not necessary to rely on concepts such as forces or efficacy, or even causation. *Séna7* can be modeled using simply quantification over stereotypical, epistemically accessible worlds. A strong hypothesis would be that *all* frustratives can be dealt with in this fashion. As Copley (2005) originally pointed out with respect to Tohono O’odham *cem:* ‘As exotic as it initially may look to English speakers, *cem* turns out to be only minimally different from other, more familiar modals.’
